# What Is Known About the Fish Intake of People Living in Disadvantaged Communities in the UK? A Scoping Review

**DOI:** 10.1111/nbu.70030

**Published:** 2025-09-21

**Authors:** Sarah Gale, Roseline Aboluwade, Louise Hunt, Clare Pettinger

**Affiliations:** ^1^ School of Health Professions, Faculty of Health University of Plymouth Plymouth UK

**Keywords:** blue food system, disadvantaged communities, fish intake, health inequalities, socioeconomic status

## Abstract

Fish provides essential nutrients, including protein, omega‐3 fatty acids (oily fish) and other micronutrients, and may be seen to have a prominent role in protecting against non‐communicable diseases, especially cardiovascular disease. Recent UK National Diet and Nutrition Survey analysis suggests people are not meeting their weekly fish intake recommendation of at least two portions per week, of which one should be oily. Lower socioeconomic groups are more likely to eat poor‐quality diets, with low fish intake, resulting in poor health outcomes. The aim of this scoping review was to examine the factors influencing fish consumption in people living within ‘disadvantaged communities’ in the UK. The review was guided by Arksey and O'Malley's five‐stage framework and Preferred Reporting Items for Systematic Reviews (PRISMA) checklist. Peer‐reviewed literature was searched, focusing on studies carried out in the UK (published in CINAHLPlus, PubMed, Scopus, Web of Science and MEDLINE) and grey literature (Google Scholar, consultations and websites) between January 2000 and December 2023. Selected studies were reviewed and analysed descriptively or using content analysis. A total of *n* = 26 papers were reviewed, with collated findings suggesting a nuanced picture in relation to fish intake within ‘disadvantaged communities’. Specific barriers were identified, including physical and economic accessibility, with poor access to fish and the high cost of fish (especially oily) positively associated with income level. Demographic characteristics of age, gender, and ethnicity were shown to influence fish intake. Education level also plays a role, namely the higher the education level, the higher the likelihood of regular (i.e., weekly or daily depending on study) fish consumption. Similarly, cultural factors can determine fish‐related food choices in adults, which can also influence children's fish intake. Despite some inherent limitations, this review provides important insights into the fish intake of disadvantaged communities. Recommendations are made for researchers, practitioners, and policy makers engaged in (blue) food system strategies to inform the design of interventions and campaigns to promote fish intake, enhance education of its health benefits, and skills in its preparation/cooking in disadvantaged communities to support action to tackle health inequalities.

## 1 Introduction

Poor nutrition and diet‐related chronic diseases in the UK are associated with socioeconomic characteristics (Hunt et al. 2023). This is exemplified by poorer quality diets and compromised nutritional intakes in lower socioeconomic status (SES) groups compared to higher socioeconomic groups (Buttriss 2019; Nelson et al. 2007; Darmon and Drewnowski 2008; Darmon and Drewnowski 2015). To reduce health and nutritional inequalities among socially and economically disadvantaged groups, there is a need to focus more on strategies to improve dietary habits. The importance of eating the right balance of nutrients has been highlighted by the recent ‘The Burden of Disease in England,’ report by Public Health England (PHE 2020a).

Fish, described as a ‘blue food’ (aquatic foods that come from oceans and waters) (Tigchelaar et al. 2022), is known to have a prominent role in protecting humans against non‐communicable diseases (Verbeke et al. 2005; Jamioł‐Milc et al. 2021). Commonly promoted to reduce the prevalence of these diseases, it has been cited as a major food within ‘healthier’ diets such as the Mediterranean diet (Willett et al. 1995; Romagnolo and Selmin 2017) and the DASH diet (Umemoto et al. 2022; Soltani et al. 2016), and is prominent within the UK's healthy eating model, the Eatwell Guide (Public Health England 2016). Several studies have examined fish consumption in the UK general population (e.g., Birch et al. 2018; Derbyshire 2019; Kranz et al. 2017) Despite the UK showing relatively stable fish eating trends over the past two decades (PHE 2020b), purchasing data show a slight downward trend of fish intake, with preference for ‘the big five’ species (cod, haddock, salmon, tuna and prawns) according to the Marine Stewardship Council (2025) report. The consumption of oily fish, however, is well below the dietary recommendations of government guidelines (SACN 2004), which encourage at least one portion per week, across all SES groups. For example, the UK's mean consumption of oily fish (56 g/week) is below the recommended level for all age groups (140 g/week) (PHE 2020b). However, few studies have specifically assessed fish intake in lower SES communities in the UK (Nelson et al. 2007), despite the increased risk of chronic diseases that disproportionately affect this sub‐population group.

### 1.1 Nutritional Benefits of Fish

The nutritional benefits of fish are well evidenced (Ruxton 2011) and include contribution to intakes of protein, omega‐3 fatty acids, and several micronutrients:

#### 1.1.1 Protein

Fish contains high‐quality protein, and due to its low saturated fat content, is often considered a healthier protein choice than meat (de Boer et al. 2020). However, this applies to fresh or minimally processed fish prepared using healthy cooking methods such as grilling, baking and steaming. Popular takeaway options such as fish and chips, often deep‐fried in batter and served with salt, can be high in saturated fat and sodium. As such, while fish is considered a healthier alternative to meat, its health benefits depend on the preparation method and context of consumption (Ruxton 2011; Mozaffarian et al. 2003). As a valuable source of protein, fish aids growth and tissue repair (Gorissen and Witard 2017). The role of fish as a protein source to tackle malnutrition (Tacon et al. 2020) and frailty (Ahn et al. 2023) in older adults means that, if cooked appropriately, it could be a beneficial replacement for other (often highly processed) protein sources and higher saturated fat meat sources (Krauss et al. 2000). However, the high price of fish as a protein source compared with some (lower quality) meat may limit its consumption for populations of any age living in lower SES communities (Darmon and Drewnowski 2015). Similarly, a sensory disliking of fish and lack of cookery skills and confidence to prepare fish have been cited as barriers to lower socioeconomic groups choosing fish as an alternative protein source (Carlucci et al. 2015).

#### 1.1.2 Omega‐3 Fatty Acids

Fish is a major source of long‐chain polyunsaturated fatty acids, eicosapentaenoic acid (EPA) and docosahexaenoic acid (DHA), known as omega‐3 fatty acids (Harris 2007). These long chain fatty acids (LC n‐3 PUFA) play an essential role in health benefits, with evidence suggesting that consumption of oily fish has an inverse effect on the risk of cardiovascular disease (CVD), attributed to its LC‐n‐3 PUFA content (Rimm et al. 2018; SACN 2004; Khan et al. 2021). Indeed, a recent meta‐analysis of 17 prospective cohort studies by Harris et al. (2021) showed unequivocally that those with the highest total levels of omega‐3 fatty acids (especially LC n‐3 PUFA) in their blood have up to a 15%–18% lower risk of dying from heart disease, compared with those with the lowest total levels of omega‐3 fatty acids. This is pertinent, especially in the UK population, where the risk of CVD is high and disproportionately affects those from the lowest SES communities (Yusuf et al. 2015).

#### 1.1.3 Micronutrients

Fish is an important source of essential micronutrients, including vitamins A, B, and D, and minerals including calcium, iron, and zinc (Tacon et al. 2020). Fish intake is also known to positively contribute to improved nutrient profiles for vitamin A (Roos et al. 2007) and vitamin B12 status (Scheers et al. 2014). Fish consumption (especially oily fish) increases concentrations of 25 (OH) D in the blood (Lehmann et al. 2015). This is particularly salient for lower SES groups, for whom the risk of vitamin D deficiency is higher (Lin et al. 2021). Oily fish is a good source of vitamin D (NHS 2020), higher intakes of which have been linked to a lower risk of CVD, autoimmune conditions, and cancers (Antico et al. 2012) all of which are also more prevalent in lower SES communities. Low vitamin D levels have been associated with reduced fish intake among lower SES groups, particularly among older adults and ethnic minority populations (Darling et al. 2018; Sutherland et al. 2021).

### 1.2 UK Disparities in Fish Consumption

Evidence suggests that lower income groups might have lower fish intake compared to higher income groups (Ruxton 2011; Bates et al. 2010), especially oily fish intake (EPA and DHA sources). For example, Nelson et al. (2007) reported mean intakes of fish to be between 84 g and 259 g per week across SES groups and that the lower income groups consumed the least. In fact, children from the lowest SES families are reported to consume 75% less oily fish than those from the highest SES families (PHE 2020b). In the low Income National Dietary Nutrition Survey (LIDNS), only 3% of adolescents and 15% of adults from low‐income households consumed oily fish (Derbyshire 2019).

Hergenrader et al. (2022) found that pregnant women from lower SES groups had lower serum levels of omega‐3 fatty acid, which could increase the risk of adverse birth outcomes such as low birth weight, stillbirths, or miscarriages. Additional studies have demonstrated a correlation between higher oily fish consumption and improved academic performance in adolescents, particularly those from lower SES groups (Lehner et al. 2020; Åberg et al. 2009).

‘Disadvantaged communities’ are widely discussed in public health research and the definition and standardisation of the term is not always clear (Maguire and Monsivais 2015). When describing the correlation between diet and socioeconomic position, it is important to consider appropriate indicators. Education level, occupation and household income are often used for this purpose and the Index of Multiple Deprivation (IMD) has become the standardised measurement (see Hunt et al. 2023). For the purposes of this scoping review ‘disadvantaged communities’ are broadly considered as being impacted by ‘material deprivation’ (DWP 2024). This is specifically defined as ‘individuals and families at risk of food and housing insecurity, often culturally diverse, who can experience multiple challenges; financial, mental health, physical health’ (FoodSEqual 2021).

Research on the fish intake of disadvantaged communities in the UK is limited. Quantitative datasets using dietary survey methodologies (e.g., National Diet and Nutrition Survey) are the predominant source of information about UK adult diets (Campbell et al. 2020) yet may misrepresent diets in disadvantaged communities because sub‐sample sizes are small (Holmes et al. 2008). While low‐quality diets, such as those containing low levels of fibre and inadequate fish, are common across the population (National Diet and Nutrition Survey), this limited quantitative evidence may mask inequalities, whereby vulnerable groups living in areas of material deprivation may be even more likely than other groups to consume lower‐quality diets, including reduced fish and fibre intakes, even in coastal communities. Whilst eating fish is widely recommended for its health benefits, it also raises environmental concerns due to global overfishing, marking it as an ecological ‘red flag’. This tension underscores how blue foods sit at the intersection of social, health and environmental inequities. For the reasons outlined above, and to inform future research and action, it is paramount to explore the available literature that assesses fish intake in disadvantaged communities, to examine consumption patterns and map knowledge gaps in the evidence.

## 2 Aim: To Scope the Available (Published and Grey) Literature to Examine the Factors Influencing Fish Consumption in People Living Within Disadvantaged Communities in the UK

This review will enable the following research questions to be answered:

- What is known about fish consumption in this UK sub‐population and how are consumption patterns associated with socioeconomic status?
- What is known about the barriers and drivers of fish consumption in this UK sub‐population group?

This scoping review was carried out as part of preliminary benchmarking activities for a national government funded consortium food system transformation project (FoodSEqual 2021). This project informs food system transformation research, policy and practice, indicating ways to improve understanding of fish consumption habits of lower SES communities, thus potentially impacting diet and health inequalities.

## 3 Methods

### 3.1 Design

Scoping reviews are essentially used to explore and map the breadth or depth of literature and to summarise evidence to inform future research (Tricco et al. 2016; Munn et al. 2022). To ensure good practice, the Preferred Reporting Items for Systematic Reviews and Meta‐Analyses—extension for Scoping Reviews (PRISMA‐ScR) checklist and guidelines outlined by Tricco et al. (2016) were employed alongside the six‐stage framework developed by Arksey and O'Malley (2005), expanded upon by Levac et al. (2010). The framework includes identifying the research question; identifying relevant studies; study selection; charting the data; and collating, summarising, and reporting the results.

Inclusion criteria stipulated studies must focus on the fish intake of people of any age living in UK ‘disadvantaged’ communities. Studies were included that sought to focus research within ‘disadvantaged communities’ across the lifecycle; were about fish (or seafood) intake of any kind (including fresh frozen, tinned, home cooked and take away, and/or supplement use e.g., vitamin D [this was because some papers focussed on fish intake as a source of vitamin D]); were available in the published literature including peer reviewed qualitative and quantitative or mixed method studies; were located within grey literature including research reports, working papers, conference papers, white papers, policy documents and theises; were written in English and published in 2000 or later. Studies were excluded if they did not take place in UK, were not representative of disadvantaged communities, were not specifically about fish/seafood intake or did not meet the inclusion criteria.

### 3.2 Published Literature Searches

The co‐researchers (two Masters students) developed search strategies after consultation with an information specialist, guided by recommendations within the JBI guides (Peters et al. 2021). Search terms were formulated by testing them across databases, and term truncations adapted for different databases. Between January 2021 to December 2023, five electronic databases were searched: CINAHL Plus, PubMed, Scopus, Web of Science and MEDLINE. For each study, a descriptive charting table was designed, informed by Peters et al. (2021), using Microsoft Excel, which included general information on the included studies and specific information relevant to the research question. These included: authors, year of publication, the study name and year of data collection, study location, aim(s) of the study, participant's characteristics (size and age group), study design, type of fish consumed, dietary assessment method, frequency/rates of fish consumption, socioeconomic measures and factors influencing fish consumption (e.g., barriers to fish consumption). Validation was carried out on a small sample (approx. 10%) of papers by an independent researcher (student supervisor) and agreement reached.

### 3.3 Grey Literature Searches

Grey literature is used within scoping reviews as an important source of relevant evidence (Adams et al. 2016). The Joanna Briggs Institute confirm that grey literature complements published literature and has the potential to reach a wider audience (Peters et al. 2021). It can be difficult to search systematically, however, as there is no ‘old standard’ for this type of search (Godin et al. 2015).

The research team created a systematic search method plan, informed by Godin et al. (2015) and Manietta et al. (2022) which included the following:

- Grey literature databases, e.g., Leeds library list of sources to access health science, social care, and government sites.
- Customised Google searches i.e., Google and Google Scholar searches to scope for studies relating to fish intake in disadvantaged communities. Simplified search strings were constructed from multiple combinations of key words based on the research question—the first ten pages were screened then exported for assessment.
- Targeted websites—the team devised a list of organisations connected to fish or fisheries and support, or that have an interest in the nutrition of low socioeconomic or disadvantaged communities in the UK. Organisation websites were hand searched for studies relating to fish intake in disadvantaged communities.
- Consultation with subject experts—The search was strengthened by contacting subject experts in the field, both local and national organisations in the UK.

### 3.4 Data Analysis

All data were appraised and collated by the research team in narrative and numerical format. Furthermore, thematic analysis was conducted to map relationships between studies and identify gaps to understand the fish consumption patterns. Research team discussions on the themes were undertaken and consensus reached to support validation.

## 4 Results

**FIGURE 1:**
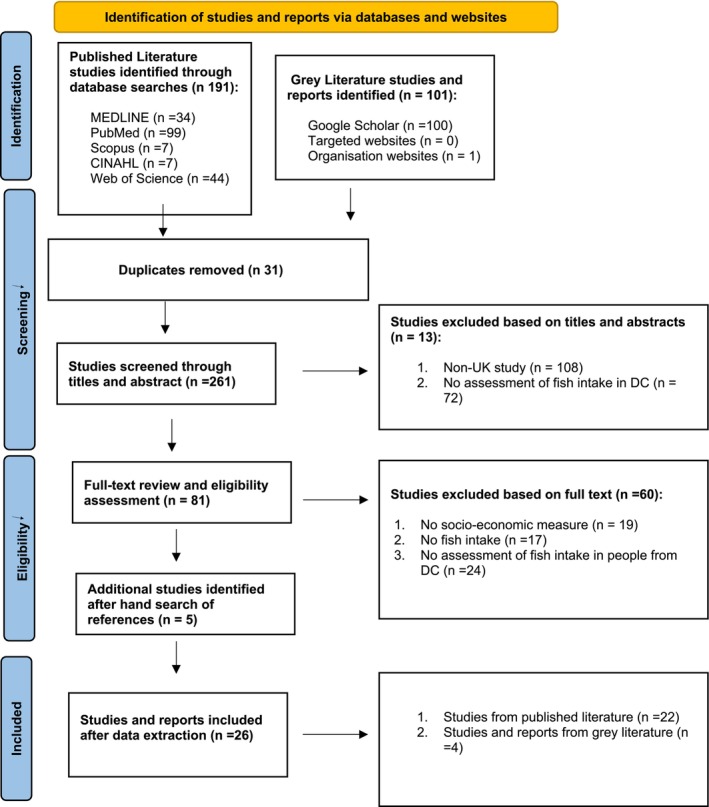
PRISMA‐ScR flow diagram showing selection process (The Joanna Briggs Institute 2018).

Records were retrieved and screened from 292 (published and grey literature) sources. Once duplicates were removed (*n* = 31) and inclusion criteria applied, a total of *n* = 266 were excluded, resulting in *n* = 26 studies being used for this review. These studies represented the views of 577 913 participants, of which at least 131 373 were from ‘disadvantaged communities’ (some studies did not provide a specific break‐down of their sample size by measure of disadvantage) (see Table 1). The PRISMA‐ScR flow diagram (Figure 1) describes the results of the search strategy and the selection process.

**TABLE 1:** Data extraction table of included studies.

Author/year of publication type of study	Study name	Data used and year of collection	Study design (type of study/dietary assessment methods used)	Sample population	Sample size	Age group	Socioeconomic measures	Type of fish consumed	Study findings summary	Factors influencing fish consumption (barriers/drivers)
1.Anderson (2007). Published literature	Nutrition interventions in women in low‐income groups in the UK	Various. A Review paper	Review of nutrition interventions for women in low‐ income groups. Mixed	Female	Various. Reviewed many studies. Total number of UK women not given.	≥ 18 years	Deprivation indices	Fish and oily fish	Women from low‐income backgrounds are more likely to eat low amounts of fish	Income, knowledge, cooking skills, price of fish, cultural norms and accessibility
2. Amuzu et al. (2009). Published literature (hand searched)	Influence of area and individual lifecourse deprivation on health behaviours: findings from the British Women's Heart and Health Study	BWHHS (1999–2001). England, Scotland and Wales	Quantitative study Longitudinal study. Dietary data—FFQ	Female	3522 (of which 634 lived in most deprived quintile)	60–79 years	IMD: 5 groups quintiles of least to most deprived and measures of lifecourse socio‐economic position.	Fish (type not explained) 67% lower deprived 76% least deprived	The intake of fish was lower among those who live in most deprived areas (67%) compared to those in the least (76%)	Socioeconomic position and environmental influence (living in an area of deprivation)
3. Barton et al. (2015) Published literature	Trends in socioeconomic inequalities in the Scottish Diet: 2001–2009 March (from UK EFS LCFS data)	Scotland (2001–2009)	Quantitative study Cross sectional 14‐day weighted food diary (from EFS/LCFS)	Household population (adults, and children adolescents)	11 374 people included in study. No data given about how many of these were from more deprived quintiles	All years	Quintiles of SIMD	Quintile 1 (most deprived) Oily fish 21g White fish 77g Quintile 5 (least deprived) Oily fish 40g White fish 112 g	Intake of oily fish and white fish per week was low in the most (21g and 77g) deprived quintile compared with the least deprived households (40g and 112g)/week	Socioeconomic position
4. Bourlakis et al. (2022). Grey literature (DSI annual conference 2022)	Mapping Food Supply Chains for UK Disadvantaged Communities: A focus on Plymouth	Plymouth, UK	Mapping of supply chains. Semi‐structured interviews	Primary producers, manufacturers, retailers, wholesalers and logistics companies, experienced academic professionals, industry experts, government officials and food charities	32 people. None were people living in disadvantage	Adults	NA	Schematic map of local fish supply chain	Little or no waste in the supply chain, resulting in no surplus food available to be redistributed to disadvantaged communities	Affordability, lack of fish processing and cookery skills
5. Darling et al. (2018). Published literature	Vitamin D supplement use and associated demographic, dietary and lifestyle factors in 8024 South Asians age 40–69 years: analysis of the UK Biobank cohort (2006–2010)	UK	Cross section analysis 24‐hour recall	South Asian M and F (Bangladeshi, Indian and Pakistani)	Of the 8024 participants, 1776 had a household income of £18 000 or under. 22% (1765) lived in most deprived Townsend deprivation scale quartile	40–69 years	Gender Ethnicity (Bangladeshi, Indian and Pakistani) Low to high intake	Oily fish Non‐oily fish	Lower household income has borderline association with being less likely to take a vitamin D supplement	Low household income
6. Emmett et al. (2015). Published literature	Pregnancy diet and associated outcomes in the Avon Longitudinal study of parents and children (ALSPAC)	Bristol and surrounding local area	Review of publications coming from a longitudinal, observational birth cohort study unquantified FFQ at 32 weeks gestation	Pregnant women and children	11,923 pregnant women. Of which 3703 had no qualification aged 16.20889 lived in council or social housing	Ages when pregnant	Educational and deprivation indices housing tenure	‐ White fish (cod, haddock, plaice, fish fingers, etc.) ‐ Dark or oily fish (tuna, sardines, pilchards, mackerel, herring, kippers, trout, salmon, etc.) ‐ Shell Fish (prawns, crabs, cockles, mussels etc.)	Higher quality diet including higher fish intake associated with mothers with higher levels of education	Low income, educational attainment
7. Fard et al. (2021). Published literature (hand searched)	On the interplay between educational attainment and nutrition: a spatially aware perspective	England, Scotland, Wales, Northern Ireland	Quant design. Household purchase data sets (Tesco‐grocery dataset)		N/R	N/R	Educational–low to high	Oily fish (canned, frozen)	A low education level is associated with a lower intake of fish	Low education
8. Food Foundation. Grey literature	2022 The Broken Plate: The State of the Nation's Food System.		Grey Literature – A document reviewing the food system		N/R				Eatwell plate (including fish) unaffordable	Low income
9. Haggarty et al. (2009). Published literature	Diet and deprivation in pregnancy	Aberdeen, Scotland 2000–2006	Quantitative study Prospective cohort study (AMND) FFQ	Female	Total sample *n* = 1461 (All levels of deprivation using SIMD). 50% from most deprived quintile *n* = 730	19–50 years	SIMD 5 groups (1st vs. 5th quintile)	Oily Fish Fish	More deprived had lower oily fish intakes. Intakes: ‐ Oily fish (*p* < 0.01) lower in the most deprived decile (particularly women) ‐ Non‐oily fish not significant	Low income, employment, housing, skills, accessibility
10. Hamer and Mishra (2009). Published literature	Dietary patterns and cardiovascular risk markers in the UK low‐income Diet and Nutrition Survey.	UK 2003–2005	Quantitative study Five‐stage clustered design (LIDNS) 24‐h recall X3	Male and Female	2721 (All participants lived in areas of deprivation using LIDNS)	≥ 16 years	IMD 3 groups: low income (less than £200/w, social housing, no qualifications, no job)	Oily fish	There was considerable heterogeneity in diets among socially deprived adults. Oily fish intake was low at 7.7 g/day	Low income
11. Hardcastle and Blake (2015). Published literature	Influences underlying family food choices in mothers from an economically disadvantaged community	Sussex, England	Prospective cohort study part of a larger study based on (Come dine with me). Qualatative: Semi structured telephone interviews	Female mothers from socially deprived area in East Sussex	16 (All from socially deprived communities)	32–49 years	IMD annual income of less than annual median income of £17, 197	Fish and chips	Fish consumption in disadvantaged families is affected by cost and budget, parental attitudes, roles of socialisation on diet and cooking skills and confidence	Cost, availability and socialisation
12. Heslehurst et al. (2023). Published literature	Maternal obesity and patterns in postnatal diet, physical activity and weight among a highly deprived population in the UK. The GLOWING pilot‐trial	North–East England (4 NHS trusts)	Quantitative study semi‐quantitative design (Secondary analysis of: Pilot cluster randomised controlled trial) FFQ	Pregnant women	163 most participants from areas of deprivation: Q1. 89 (most deprived) Q2. 36 Q3. 14 Q4. 15 Q5. 8 (Least deprived) Missing 1	Mean age 29.2 years	‐ IMD ranks grouped into quintiles of equal proportion to determine deprivation status ‐ Quintile 1 was the most deprived and quintile 5 was least deprived	All fish including processed and oily fish	Consumption of fish is primarily from processed sources. Intake of unprocessed fish is low (16–37 g/day), specifically oily fish (0.4 g/day)	Deprivation, cost and education
13. Holmes et al. (2008). Published literature	How access, isolation and other factors may influence food consumption and nutrient intake in materially deprived older men in the UK	UK 2003–2005. 24‐hour recall (multiple)	Quantitative study Cross sectional (LIDNS) 24‐hour recall (multiple)	Older Adults (Male)	234 (All participants lived in areas of deprivation using LIDNS)	≥ 65 years	IMD	Fish	Deprived older men living in households where the main food provider in the household had better cookery skills consumed more fish and fish dishes 22 g/day vs. less cooking skills (12 g/day)	Social factors (accessibility, affordability, choice and variety), Physical factors (mobility, oral health, ability to shop and prepare fish)
14. Holmes and Roberts (2011). Published literature	Diet quality and the influence of social and physical factors on food consumption and nutrient intake in materially deprived older people	UK 2003–2005	Quantitative study Five‐stage clustered design (LIDNS). Multiple 24‐hour recalls	Male and Female	662 (all participants lived in areas of deprivation using LIDNS)	≥ 65 years	IMD	Whitefish and Oily fish	Daily consumption of white fish and oily fish for older deprived women in the best diet group (28g and 12g) was more than that of older deprived men (17g and 11g). Sociability associated with better diet including higher levels of fish intake	Accessibility, affordability and choice. Sociability (eating with others) associated with better diet including more fish
15. Jones and Chikwama (2021). Published Literature (hand searched)	Access to marine ecosystems services: Inequalities in Scotland's young people. *Ecological Economics*, *188*, p.107139	Scotland 2016	Quantitative study. Self‐administered FFQ	Adolescents	1550 in total. The number coming from higher levels of deprivation (by SMID) not provided	11–16 years	SIMD (most vs. least deprived) quintile groups	Fish and shellfish	Inequality in consumption – those living in more deprived places less likely to consume fish and seafood then those living in less deprived places	Area level deprivation
16. Kranz et al. (2017) Published literature	Intake levels of fish in UK paediatric population	UK	Quantitative study. NDNS‐RP, 2008–2012. Food diaries (4 days)	Children	2096 (of these, 440 lived in social housing)	2–18 years	Socio demographic data using income	Total fish, white fish, oily fish, canned tuna, and shellfish	Logistic regression models showed that neither household income nor ethnic group was associated with total fish or oily fish intake– NDNS	No association of fish intake with household income for children
17. Maguire and Monsivais (2015). Published Literature (hand searched)	Socioeconomic dietary inequalities in UK adults: an updated picture of key food groups and nutrients from national surveillance data	UK 2008–2011	Quantitative study Cross sectional study (NDNS) Food diary (4 days)	Adults	1491 (of these 339 were from the lowest two occupational groups, while 634 were from the highest two)	≥ 19 years	High Income, Occupational class and Education levels (Highest v lowest socioeconomic groups)	Oily fish	Oily fish intake increased by income and education level	Socioeconomic position, most especially for women, education level and occupation
18. Mesirow et al. (2017) Published literature	Associations between parental and early childhood fish and processed food intake, conduct problems and co‐occurring difficulties	South–West of England	Quantitative study Longitudinal study FFQ	Mothers and children	5727 mother/child pairs (of these only 4.1% represent women from ethnic minorities). Does not say how many form low SES	Prenatal, 3 years; 4–10 years; 12–13 years	Family adversity index (FAI)	3 fish items of white, oily and shellfish and processed or non‐processed	Finds low SES and more family adversity more likely to have conduct problems	Low socioeconomic backgrounds
19. Nelson et al. (2007). Grey literature (nutrition survey‐ government report)	Low‐income Diet and Nutrition Survey (LIDNS)	UK	Quantitative study Five stage clustered design 10‐day 4 × 24‐hour recall	Children, adolescents and adults	3728 (all participants living in material deprivation)	≥ 2 years	IMD	Oily and white fish	Only 15% of adults ate oily fish, with an overall 34 g/w for men and 48 g/week for women. For total fish, children 74 g/week and adolescents 84 g/week, adults 112 g/w and older adults 126 g/week. Low income associated with eating low amounts of fish	Low income
20. O'Neill et al. (2004) Published literature (hand searched)	Barriers to healthier eating in a disadvantaged community	Wales, Merthyr Tydfil	Qualitative (Participatory action research). Focus groups	Adolescents, adults	115 (all participants were living in the most deprived area in the UK)	≥ 18 years	WIMD	Fish, fish and chips	Older adults placed fish in the first position for healthiest food, while other age groups placed fish in the last. Commonly consumed fish is deep‐fried fish and chips	Time, resources, taste, inconsistent messages from professionals
21. Ruxton (2011). Grey literature (review of nutrition surveys)	The Benefits of Fish Consumption Review	UK	Review on UK survey data (NDNS and LIDNS) 24‐h recall over 4 days	Children, adolescents, and adults	Various (including data from low‐income adults/adolescents using LINDS)	All ages	Deprivation indices	Oily and white fish	‐To increase fish consumption‐suggests Improving understanding of 2‐a‐week message (anomalies in portion sizes)	High cost of fish, poor cookery skills, lack of confidence in selection of all types of fish (fresh, frozen, convenience fish meals)
22. Sutherland et al. (2021) Published literature	Differences and determinants of vitamin D deficiency among UK biobank participants: A cross‐ethnic and socioeconomic study	England, Scotland, and Wales	Quantitative study UK Biobank large scale prospective cohort study Questionnaire surveys, FFQ, 24 h recall	Male and Female White European Chinese Asian Black African Mixed ethnicity	502 316 (of these, one quarter, 110 010, were from the lowest quarter of the Townsend index)	40–69 years	Townsend deprivation index	Oily fish	Low income associated with higher prevalence of vitamin D deficiency for all ethnic groups except Black Africans. Low education associated with deficiency for Asians but not for other ethnicities	Affordability of vitamin D supplements and therefore perhaps also fish
23. Tong et al. (2018). Published literature	Dietary cost associated with adherence to the Mediterranean diet and its variation by socioeconomic factors in the UK fenland study	Fenland Study from general practices in Cambridgeshire	Quantitative study. Population‐based cohort study 130‐item semi‐ FFQ to calculate a Mediterranean diet score (MDS)	Male and Female	12,435 (comparison of 3 areas: Cambridge, Ely, Wisbech from least to most deprived based on household income)	30–65 years at recruitment	Education level, marital status, occupation and household income.	Fish	First study to demonstrate the extent to which socioeconomic factors (education, income and occupation) contribute to the association between a Mediterranean diet, including fish, (MD) and dietary cost	Cost of fish
24. Whybrow et al. (2018). Published literature	Social deprivation is associated with poorer adherence to healthy eating dietary goals: analysis of household food purchases	Scotland	Quantitative study. Cross‐sectional	Male and Female	2586 (Household data using SIMD: Q1 439 (most deprived) Q2 571 Q3 565 Q4 543 Q5 468) (least deprived)	2–59 years	SIMD	Oily fish	Households in the most deprived areas had low fish intake (2.7 g/day) compared with the least deprived	Availability (quality of fish available)
25. Wrieden et al. (2004). Published literature	Secular and socioeconomic trends in compliance with dietary targets in the north Glasgow MONICA population surveys 1986–1995: did social gradient widen?	Scotland, North Glasgow	Quantitative study. Cross‐sectional FFQ	Male and Female	5624 (all participants living in areas of social deprivation and 40% of sample in most deprived DEPCAT score 7)	25–64 years	SIMD (least vs. most deprived)	Oily fish	Gender 38% men 44% women ate fish once a week all years (1986–1995); by deprivation, there was an overall change from 30% in 1986 to 42%–47% in 1995 in both least and most deprived	Low income (income support not enough to afford healthy food i.e., fish), affordability, accessibility and cookery skills
26. Wrieden et al. (2007). Published literature	The impact of a community‐based food skills intervention on cooking confidence, food preparation methods and dietary choices‐ an exploratory trial	Scotland	Quantitative study Quasi‐experimental design FFQ and 7‐day food diaries	Male and Female	113	≥ 18 years	SIMD	White, oily fish, tuna, shellfish	At baseline, fish consumed a mean of once a week (tuna between half to a third of this). After the intervention, no significant changes were observed	Cooking confidence

Abbreviations: AMND, Aberdeen Maternity and Neonatal Database; AYPSS, Annual Young People in Scotland Survey; BWHHS, British Women Health and Heart Study; EFS, Expenditure and Food Survey; FFQ, Food Frequency Questionnaire; IMD, Index of Multiple Deprivation; KWHFP, Kantar Worldpanel Household Food Purchase; LCFS, Living Costs and Food Survey; LIDNS, Low Income Diet and Nutrition Survey; MFP, main food provider; MONICA, Monitoring Trends and Determinants in Cardiovascular Diseases; N/R, not reported; NDNS, National Diet and Nutrition Survey; SIMD, Scottish Index of Multiple Deprivation; TGD, Tesco Grocery 1.0 Dataset; WIMD, Welsh Index of Multiple Deprivation.

Table 1 Data extraction table, which includes all the key information relevant to the studies selected and the aim of the scoping review. This includes a description of the key findings from the selected studies.

### 4.1 Summary of (Demographic) Findings

The age range for participants within the reviewed studies was 2–79 years. Four studies included children, six studies included adolescents, eighteen studies included adults and older adults of both sexes, four studies included only women, one study included only men, and two studies included adults from minority groups.

All studies were conducted within a variety of UK geographical locations. Nine studies (Anderson 2007; Amuzu et al. 2009; Darling et al. 2018; Fard et al. 2021; Hamer and Mishra 2009; Holmes et al. 2008; Maguire and Monsivais 2015; Nelson et al. 2007; Sutherland et al. 2021) recruited participants from across the UK, which represented a wider distribution of the population. Six studies (Barton et al. 2015; Haggarty et al. 2009; Jones and Chikwama 2021; Whybrow et al. 2018; Wrieden et al. 2004; Wrieden et al. 2007) were conducted in Scotland. The Food Foundation (2022) study used a dataset from Tesco's stores across Greater London; Hardcastle and Blake (2015) recruited mothers from socially deprived areas in East Sussex, England, and O'Neill et al. (2004) targeted Merthyr Tydfil, Wales.

The sample sizes ranged from *n* = 16 (Wrieden et al. 2004; Hardcastle and Blake 2015) to *n* = 502 316 (Sutherland et al. 2021) reflecting the variation in study designs.

The smaller sample sized studies (e.g., Wrieden et al. 2004) aimed to evaluate the feasibility of altering food choices, cookery skills, and food preparation in people from deprived areas, including measurement of frequency of fish consumption. One study (Hardcastle and Blake 2015) consisted of 16 female mothers from socially deprived areas in East Sussex, and O'Neill et al. (2004) study questioned 115 adolescents and adults about barriers to healthier eating. The larger studies included investigation of the UK Biobank prospective cohort study (Sutherland et al. 2021), the National Diet and Nutrition Survey (NDNS) (Maguire and Monsivais 2015) and the LIDNS (Haggarty et al. 2009).

All studies provided some information about fish eating in areas of deprivation/disadvantage, although not all studies provided a breakdown of their sample size by measure of disadvantage, meaning some findings need to be interpreted with caution. A range of socioeconomic measures were used to define disadvantage. Most studies (i.e., 20 of the 26) used area‐based deprivation measures which included Index of Multiple Deprivation (IMD), the Scottish Index of Multiple Deprivation (SIMD) or the Welsh Index of Multiple Deprivation (WIMD). These area‐based measures of deprivation, from least to most deprived, utilised measures of household income, employment, education, health, crime, and housing. The Food Foundation (2022); Jones and Chikwama (2021); Maguire and Monsivais (2015); and Nelson et al. (2007) focused on specific indicators such as education levels; these were categorised by educational qualifications, vocational qualifications, and type of school or degree qualification, whilst Darling et al. (2018) and Sutherland et al. (2021) focused on ethnicity indicators.

### 4.2 Dietary Assessment Measures of Fish Intake

Food frequency questionnaires (FFQ) were used by some studies to report fish type and the amount and frequency of fish/seafood consumption (Amuzu et al. 2009; Wrieden et al. 2004; Wrieden et al. 2007). One study excluded shellfish (Wrieden et al. 2007). Others used 24‐h recall methods whereby the type, amount, and frequency of consumption were examined (Darling et al. 2018; Hamer and Mishra 2009; Holmes et al. 2008; Nelson et al. 2007). Weighed food diaries were also used, which requested fish consumption information differentiated by white and oily fish (Haggarty et al. 2009). Other studies used food diaries of up to 14 days (Barton et al. 2015; Fard et al. 2021; Maguire and Monsivais 2015). Food diary data from large data sets were also employed, e.g., Scottish sample of food purchase data from the UK Expenditure Food Survey (EFS) and Living Costs and Food Survey (LCFS) (Barton et al. 2015) or the NDNS 2008–2011 (Maguire and Monsivais 2015). These cases recorded the type and amount of consumption and applied linear regression analysis to track trends in lower socioeconomic groups.

Some of the smaller qualitative studies used semi‐structured telephone or group interviews to examine fish intake. For example, they used group interviews to establish the barriers to healthy eating (including fish consumption) (O'Neill et al. 2004). Finally, some studies used food purchasing data to gather information, for example, grocery stores in Greater London (Fard et al. 2021).

### 4.3 Types and Amounts of Fish Consumed

The types of fish recorded by the included studies were white fish, oily fish, shellfish, canned fish, and take‐away fish (e.g., fish and chips). These were quantified in different ways, including the amount of fish eaten in grams per week/day or of consumers within population groups (socioeconomic group, sex, ethnic group) (Table 1). Data from 11 studies presented the frequency of fish intake by households, age groups, ethnic groups, and areas of deprivation (Amuzu et al. 2009; Barton et al. 2015; Darling et al. 2018; Hamer and Mishra 2009; Heslehurst et al. 2023; Holmes et al. 2008; Holmes and Roberts 2011; Jones and Chikwama 2021; Nelson et al. 2007; Whybrow et al. 2018; Wrieden et al. 2004). By area of deprivation, one study showed fish intake frequency was higher in the least deprived areas compared to the most deprived areas; e.g., 76% of adults from the least deprived areas reported eating fish more than once a week compared to 67% of adults from the most deprived areas (*p* < 0.0001) (Amuzu et al. 2009).

### 4.4 Thematic Findings

Four key themes were identified during the extraction and analysis of the reviewed papers: (1) Age and sex; (2) Ethnicity, cultural norms, and habits; (3) Education (nutritional knowledge and cookery skills); (4) Accessibility: (a) Physical accessibility (food environment) and (b) Economic accessibility (cost and affordability).

### 4.5 Theme One—Age and Gender

Findings from two studies indicate that older adults (over 60 years) recognise fish as a healthy food, with data demonstrating a positive correlation between age and frequency of fish consumption (Nelson et al. 2007; O'Neill et al. 2004). Meanwhile, Holmes and Roberts (2011) report that older women were found to consume greater amounts of both oily fish (12 g/day) and white fish (28 g/day) compared to older men (11 g/day and 17 g/day, respectively). Nelson et al. (2007) reported the highest mean weekly intake of oily fish among older adults (126 g/week), followed by adults (112 g/week), adolescents (84 g/week), and children (34 g/week). In contrast, the lowest recorded individual intake was 7.7 g/day in adults (Hamer and Mishra 2009). Younger age groups were more likely to perceive fish as unhealthy, primarily due to associations with fast food such as fish and chips (O'Neill et al. 2004). The Low‐Income Diet and Nutrition Survey (LIDNS: *n* = 3728) found that only 3% of children (*n* = 932) and 15% of adults (*n* = 2796) reported consuming fish during any 24‐h recall period (Nelson et al. 2007). However, the picture is nuanced, and socioeconomic disparities were also evident: older males from lower socioeconomic backgrounds were less likely to prepare or consume fish dishes (Holmes et al. 2008), and most health behaviours, including fish consumption, in older females were found to be associated with the deprivation level of the area in which they lived (Amuzu et al. 2009).

Sex‐based differences in oily fish consumption have been reported, with several studies indicating higher intake among females. Wrieden et al. (2004) found that 50% of females consumed oily fish compared to 42% of males, with an overall increase in weekly consumption between 1986 and 1995. Similarly, Sutherland et al. (2021) reported slightly higher prevalence among females (18.5%) than males (17.5%), though the difference was minimal and unrelated to socioeconomic deprivation. In adolescents, Jones and Chikwama (2021) found a non‐significant difference (*p* = 0.067) in fish intake between boys (90.4%) and girls (86.5%) with no clear link to deprivation. Additionally, one study (Darling et al. 2018) noted that women from Bangladeshi, Indian, and Pakistani backgrounds were more likely than men to report never consuming oily fish. Furthermore, Nelson et al. (2007) reported slightly higher daily intake among females (7 g/day) compared to males (5 g/day), though estimates may be inflated due to the inclusion of composite dishes.

### 4.6 Theme Two—Ethnicity, Cultural Norms and Habits

Studies examining ethnic differences in fish consumption revealed notable patterns, though these findings should be interpreted cautiously due to limited and variable data. For instance, Emmett et al. (2015) reported minor variations in the prevalence of fish intake among ethnic groups, with 31% of Pakistani, 28% of Bangladeshi, and 26% of Indian participants reporting regular consumption. Sutherland et al. (2021) found that 31% of Black African participants consumed oily fish at least once a week, compared to 19% of Chinese and 15% of South Asian participants. This study, which focused on vitamin D deficiency, a condition closely linked to oily fish intake, highlighted that low income was associated with a higher prevalence of deficiency across all ethnic groups except Black Africans. Furthermore, low educational attainment was associated with a higher prevalence of deficiency among Asian participants, but not among other groups.

Qualitative data, though limited, further reported the role of cultural and social influences on dietary behaviours. One study (O'Neill et al. 2004) indicated that cultural practices and norms shaped food choices, while another (Hardcastle and Blake 2015) identified that preferences for commonly consumed fish products, such as fish fingers, were influenced by cultural and social norms.

### 4.7 Theme Three—Education (Nutrition and Cookery Skills)

Educational status was consistently associated with fish intake across some of the studies. Emmett et al. (2015) specifically investigated the association between education and fish intake and found that higher maternal educational attainment was associated with better overall diet quality during pregnancy, including a greater likelihood of fish consumption, which in turn was linked to better child cognitive outcomes, whilst women with lower education were less likely to eat fish. Similarly, Maguire and Monsivais (2015), Jones and Chikwama (2021) and Nelson et al. (2007) reported a positive correlation between education level and fish consumption.

Evidence also suggests that cookery skill interventions may influence dietary behaviours, particularly in disadvantaged populations. Two studies (Hardcastle and Blake 2015; Wrieden et al. 2007) highlighted the value of community‐based and family‐oriented interventions in improving cooking confidence among disadvantaged groups. Wrieden et al. (2007) evaluated a cooking skills programme delivered in eight deprived communities and reported increased confidence in following recipes, though no significant change in fish intake was observed. Similarly, Hardcastle and Blake (2015) found that a school‐based intervention where children acted as health messengers increased mothers' confidence in preparing healthy meals and revealed that cost, cultural norms, and limited skills influenced food choices. While neither intervention directly targeted fish consumption, both studies suggest that building cooking confidence and engaging families can support healthier eating practices in socioeconomically disadvantaged settings. Furthermore, Bourlakis et al. (2022) and Ruxton (2011) identified limited cooking skills and poor understanding of how to prepare fish as significant barriers to consumption, particularly among individuals in disadvantaged areas.

### 4.8 Theme Four—Accessibility

#### 4.8.1 Physical Accessibility (Food Environment)

One study reported that a higher density of fast‐food outlets influenced the food choices of lower income, unmarried younger adult males, who were more likely to be exposed to unhealthy fast food dietary patterns instead of eating more expensive and less available healthier foods (Hamer and Mishra 2009). A study comparing coastal to non‐coastal locations (Jones and Chikwama 2021) reported that children are more willing to try seafood in non‐coastal locations. In this study, which analysed inequality using the Scottish Index of Multiple Deprivation (SIMD), half of the young people had never tried shellfish but inequalities in willingness to try were not observed. As well as cost (see below), other barriers including poor access and food choices resulted in lower fish intake in deprived groups (Anderson 2007; Holmes et al. 2008). A study analysing the impact of isolation and accessibility on food consumption revealed that older men with physical disabilities were less likely to consume fish (Holmes et al. 2008). Furthermore, Hardcastle and Blake (2015) and Wrieden et al. (2004) highlighted limited accessibility and availability of ‘quality’ fish as barriers to consumption.

#### 4.8.2 Economic Accessibility (Cost and Affordability)

Economic access has been identified as a significant barrier to oily fish consumption. Maguire and Monsivais (2015) reported that low income was associated with reduced oily fish intake. These findings align with Tong et al. (2018), which identified dietary cost as a barrier to adherence to Mediterranean diets, including fish consumption. Several studies have further highlighted that low income and occupational status are directly associated with lower fish intake. For example, Mesirow et al. (2017) examined poor nutrition in early childhood and found that maternal intake of healthy foods, including fish, was inversely associated with consumption of processed foods and early onset behavioural problems in children. This study suggested that low household income contributed to reduced fish intake among pregnant women and children, impacting overall dietary quality. Anderson (2007) suggested that women living in households in receipt of benefits were less likely to have consumed oily fish than their more affluent counterparts. Similarly, Amuzu et al. (2009) reported the prevalence of fish intake to be significantly lower (*p* < 0.0001) in women living in the most deprived areas. The same study also found that 76% of adults from the least deprived areas reported eating fish more than once a week compared to 67% of adults from the most deprived areas (*p* < 0.0001). Barton et al. (2015) reported the most deprived quintile of participants ate a mean of 20.8 g oil‐rich fish and 77.2 g white fish per week compared to the least deprived who consumed 31.2 g and 98.5 g per week (*p* < 0.001 and *p* = 0.008 respectively). Similarly, lower earnings were linked to lower fish intake, with Maguire and Monsivais (2015) showing a large proportion of participants (*n* = 1491 adults) ate no oily fish (72.2%), but all higher socioeconomic groups were more likely to eat oily fish than lower income groups, with an odds ratio of 4.0 for the highest income participants and a gradient in odds across the income groups. Furthermore, two studies, Emmett et al. (2015) and Heslehurst et al. (2023), reported that pregnant women from the most deprived areas and lower income households consumed less fish compared to those in less deprived areas and higher income groups. Similarly, Bourlakis et al. (2022) identified affordability as a major barrier to fish consumption in disadvantaged communities. However, Kranz et al. (2017) found no association between children's fish intake and household income.

## 5 Discussion

The purpose of this scoping review was to examine the published and grey literature to find out what is known about fish consumption in people living in disadvantaged communities in the UK. The main questions asked were: (i) what is known about fish consumption in this UK sub‐population and how are consumption patterns associated with socioeconomic status? and (ii) what is known about the barriers and drivers of fish consumption in this UK sub‐population group?

Through comprehensive review of *n* = 26 papers, representing 577 913 (of these, at least 131 373 were living in areas of deprivation/disadvantage) participants across the lifecycle, collated findings suggest a nuanced picture in relation to the fish intake of people living in disadvantaged communities. Study designs were variable, as were the dietary assessment methods used (see limitations section). Nonetheless, the review suggests that low SES and area‐level deprivation shape the fish‐eating patterns of disadvantaged communities with people from lower SES groups being more likely to be non‐fish consumers and eating less fish or seafood than their higher SES counterparts.

According to thematic findings, there was some evidence that age, sex, and ethnicity (to some degree) were shown to influence fish intake. Similarly, education level and cultural factors play a role. Physical and economic accessibility were notable factors, with poor access to quality fish (i.e., options other than take away fried fish) and the high cost of fish (especially oily) being associated with income level within the studies.

### 5.1 Age‐Related Fish‐Eating Factors in Disadvantaged Communities

Age and fish intake were considered by several of the included studies (Table 1) with older adults (over 60 years) being the group that exhibited the highest consumption of fish. However, nuance was noted, highlighting that age can interact with barriers such as accessibility, social isolation (Tani et al. 2023), limited access to shops, and poverty. Overall, there seems to be reasonable evidence to confirm that area‐level deprivation is important and that fish/seafood consumers are more likely to be older and more affluent, as confirmed by a recent systematic review of the determinants of seafood consumption in high‐income countries by Govzman et al. (2021). The included studies, which focussed on older people (> 60 years) (i.e., Amuzu et al. 2009; Holmes et al. 2008; Holmes and Roberts 2011) support research on the benefits of eating fish to prevent age‐related diseases such as CVD (Martínez‐González et al. 2019; Harris et al. 2021) due to the biologically active compounds in fish (omega −3 PUFAs, vitamin D, proteins). Indeed, a study by Bakre et al. (2018) supports the strength of socioeconomic inequalities in relation to the determinants of fish consumption in older adults (this study was excluded from the scoping review because it was not UK resident focussed). There is additional research on the positive benefits of fish eating in older adults, linking this to alleviation of memory loss and cognitive decline (Morris et al. 2005; Noyeens et al. 2018), possible dementia prevention (Tsurumaki et al. 2019) and potentially aiding prevention of sarcopenia (Rondanelli et al. 2020; Dorosty et al. 2016) further supporting the need to understand and promote fish intake, although this was beyond the remit of this scoping review.

The limited and somewhat inconsistent evidence reported here points towards the need for further research to better understand the determinants of fish intake in older (disadvantaged) adults to support action and policies for fish/dietary recommendations for this age group (BNF 2024) to improve health outcomes.

### 5.2 The Role of Sex in Relation to Fish Intake

The reviewed studies suggest that in general, more females eat fish than males (Holmes and Roberts 2011; Nelson et al. 2007; Wrieden et al. 2004), which is somewhat unsurprising given the strong evidence supporting females as more nutrition and health aware than males (Feraco et al. 2024; FSA 2022). Studies over the past few decades have shown consistent demographic (sex) variation in nutritional knowledge and food choice behaviours, and this difference tends to be more marked in lower SES groups (Parmenter et al. 2000; Wardle et al. 2004; Corfe 2018), although interestingly, in Kranz et al.'s (2017) exploration of children's fish‐eating habits, no association between fish intake and household income was found. Govzman et al. (2021) did not find any sex differences in seafood consumption, which concurs with a study of young adults' meat and fish intake by Frąckiewicz et al. (2023) who found no statistically significant sex differences in fish intake. This supports the need for interventions focussing on increasing fish and seafood intake across both sexes.

### 5.3 Ethnicity, Deprivation and Fish‐Eating Behaviours

As highlighted in the findings, trends were apparent that suggest variability between and among ethnic minority groups in relation to fish intake, especially oily fish (Darling et al. 2018; Sutherland et al. 2021), but these studies were not linked specifically to deprivation and/or only a small proportion of the sample was deprived, so caution is required when interpreting these results. Deprivation levels across UK ethnic minority groups are known to be marked, as reported by the Commission on Race and Ethnic Disparities Research (Gov.UK 2021). As most national food/nutrition datasets are not representative of minority groups, there is limited robust data on their food intake (Bennett et al. 2022). This accentuates health inequity (Marmot 2020) and makes interpretation of fish data difficult. This suggests the need for future research to explore the lived experience of ethnic groups to enable better understanding of the cultural context of their (fish) eating behaviours.

### 5.4 Cultural (and Family) Influences on Fish Intake

Cultural factors were found to significantly influence dietary behaviours in several studies. For example, Hardcastle and Blake (2015) demonstrated that strong cultural norms and socialisation contributed to the intergenerational transmission of food choices, from parents to children. One family reported regularly consuming fish and chips, despite recognising it as unhealthy according to public health advice.

Similarly, a further study, O'Neill et al. (2004), highlighted how cultural traditions and established family habits influenced food preferences, with some Welsh participants perceiving fish as unhealthy due to its association with takeaway preparation methods. These perceptions reflect a disconnect between public health guidance, such as the UK government's ‘Eatwell Guide’ (PHE 2016), and culturally embedded dietary practices. For low‐income groups, adherence to such guidelines may be further hindered by socioeconomic constraints, contributing to experiences of shame and stigma associated with not meeting public health expectations (Scott et al. 2018). Historical evidence has shown that deep‐fried fish and fried fish sandwiches are associated with stroke, heart disease (Mozaffarian et al. 2003) and cardiovascular mortality risk. In contrast, evidence has found that oily fish is particularly beneficial for the cardiovascular system (Harris et al. 2021; Mohan et al. 2021). Notwithstanding, socio‐cultural preferences play a vital role in food choice and implications for healthy diets (Monterrosa et al. 2020) and this review suggests the cultural context of fish intake is as important as its health benefits when it comes to promoting fish intake in disadvantaged communities.

There is historical evidence indicating that past behaviours and attitudes continue to influence fish consumption patterns (Honkanen et al. 2005). A study by Carstairs et al. (2017) highlights the role of the mother's personal history and relationship with fish in shaping her decisions about encouraging seafood consumption in her children. This corroborates that individuals in the most deprived areas are less likely to consume seafood if the behaviour was not established during childhood (Jones and Chikwama 2021). Additionally, the importance of seafood intake in children has been further emphasised by recent research demonstrating its positive effects on prosocial behaviour (Nel et al. 2025). Future action and policies are needed to promote fish intake to lower‐income consumers which more fully consider ethnicity, culture, and habits as key contributing factors that could influence the success of future campaigns and/or interventions.

### 5.5 Education Level and Its Association With Fish Intake

The reviewed studies uncovered a clear relationship between the level of education and the frequency or amount of fish consumed. Education is a key domain in the Townsend Index of Multiple Deprivation list and a measurement used in many of our studies (Hardcastle and Blake 2015; Hamer and Mishra 2009; Jones and Chikwama 2021; Nelson et al. 2007; Sutherland et al. 2021; Wrieden et al. 2007). As the results show (Table 1), the higher the education and occupational level, the higher the likelihood of a person to consume fish. One study (Fard et al. 2021) used a supermarket dataset to observe how education influenced healthy food purchases, stating clearly how higher education correlated to more nutritional diversity, which was characterised by eating diets higher in fish, fibre, and fruits and vegetables. This is also supported in the literature whereby the higher education level is positively correlated with fish intake (Marinac Pupavac et al. 2022) and lower education level is associated with low fish intake or poorer quality fish e.g., fried (Zhu et al. 2023). In fact, Maguire and Monsivais (2015) reported that a person with a degree was three times more likely to eat quality (oily) fish than a person with no qualifications, thus oily fish consumption increased by education and occupational level, suggesting the need for urgent public health campaigns to improve UK intakes (Derbyshire 2019). This is already part of the UK's policy strategy (DEFRA 2022) to understand better through research how to optimise promotion and increase UK fish/seafood consumption.

### 5.6 Lack of Skills to Prepare and Cook Fish

A key barrier to fish consumption identified in this review has been identified as inadequate cooking and preparation skills, and lack of confidence. These issues are often more pronounced in deprived communities where access to food education and resources is limited (Anderson 2007; Bourlakis et al. 2022; Fard et al. 2021; Holmes et al. 2008; Ruxton 2011; Wrieden et al. 2007). Several of these studies recommended improving knowledge and skills related to fish preparation, particularly in deprived communities. Targeted research and interventions addressing this barrier may support increased fish and seafood intake (DEFRA 2022). Hardcastle and Blake (2015), for example, highlighted the value of parent–child interventions in changing cooking habits and improving nutrition knowledge. This supports the role of community‐based programmes and interventions that promote fish cookery skills among children (Huss et al. 2013; Garcia et al. 2016; Mahmudiono et al. 2020). The wider literature corroborates this by promoting fish nutrition education interventions to support understanding of national fish intake recommendations in children and young people (Utri‐Khodadady et al. 2024). For example, knowledge and attitudes towards fish were shown to improve in school children in a randomised control trial incorporating education to increase fish intake (Mahmudiono et al. 2020). What's more, the type of education (pedagogy) might also be important as a qualitative study on ‘slow fish education’ showed positive results to support better understanding in children (Lee 2022). The Wrieden et al. (2007) study reported a prominent focus on the role of food skills interventions to improve food choice and confidence in food preparation (including fish), but this study suggests measures of cooking skills confidence present ongoing challenges for study design (see Lavelle et al. 2017). Some other studies exploring cooking skills have suggested low confidence in cooking fish, especially oily fish (Adam et al. 2015). Similarly, relating to the age/gender theme above, children might have low fish/seafood intake due to lack of parental cooking confidence (Burns et al. 2024). All of this supports the need for practical educational and skills interventions to tackle these inequities and promote fish intake.

### 5.7 Educational Strategies to Increase Fish Consumption

Some research postulates that interventions aimed at improving eating behaviours are less successful within lower SES groups (Attree 2005). Yet our reviewed studies suggest small successes aimed at reducing health inequalities can work well being clustered geographically in localised deprived areas (e.g., study 1). Furthermore, such small‐scale evidence can inform intervention design and delivery (e.g., study Wrieden et al. 2007). In terms of how to use educational strategies to influence increased fish consumption in disadvantaged populations, research has long shown that increasing knowledge alone is not enough to change behaviour (Parmenter et al. 2000; Worsley 2002; Achterberg and Miller 2004) and that the wider factors, driven by socio‐cultural values, need to be considered utilising transdisciplinary collaborations (Hunt et al. 2023) to support implementation. When it comes to promoting fish intake specifically, recent evidence suggests that diverse food system stakeholders, for example supply chain workers, also need to be educated to improve (low income) consumer awareness of local fish and how to prepare them (Koehn et al. 2020). The ability to upskill people to understand, prepare, and cook fish and seafood, across the supply chain, is an important recommendation—more interventions targeted towards lower socioeconomic communities would be a springboard for further improvements in this area.

### 5.8 Physical Accessibility to Fish (Food Environment)

This scoping review was limited in its findings to show whether people from disadvantaged communities choose to eat less fish because of physical accessibility or environmental barriers. Several reviewed studies (Anderson 2007; Food Foundation 2022; Holmes et al. 2008; Jones and Chikwama 2021; Kranz et al. 2017) reported generic findings of relevance whereby living in deprived neighbourhoods meant reduced physical access to shops with affordable quality healthy food items. Although evidence of the impact of a such limited food environments on food choices (Anderson 2007), and in turn, on health (Amuzu et al. 2009) was provided, no study touched on physical accessibility in relation to fish and seafood specifically, which perhaps identifies an important gap in the literature—the fish environment as a driver/barrier. It is well evidenced that the food environment plays a role in (un)healthy food access (Williamson et al. 2017). This impact is evident in retail (Macdonald et al. 2009) and across the supply chain (Drejerska and Sobczak‐Malitka 2023), however the marked heterogeneity of the study designs measuring ‘lack of healthy food’ (Titis et al. 2021) make findings difficult to interpret. Jones and Chikwama's (2021) examination of how the marine environment might influence fish consumption, confirmed marked inequalities with young people living in deprived areas being less likely to consume a range of seafood than their less deprived counterparts.

Physical accessibility to consuming fish and seafood may not present a barrier, but it certainly seems to be linked to supply chain aspects. Bourlakis et al.'s (2022) focus was on supply chain enablers to impact consumers' intake of fish and seafood. The authors claimed that physical access is not the major challenge; instead, they confirm some of the barriers already highlighted, e.g., lack of consumer skill to prepare and cook fish (Neale et al. 2012). In fact, the biggest challenge they identified was affordability of and economic access to fish, which is supported by Koehn et al. (2020), who stipulate that negotiating price points (policy) within supply chains is important to impact cost for (low income) consumers.

### 5.9 Economic Accessibility to Fish

#### 5.9.1 Affordability

Economic accessibility and affordability of buying fish and seafood were identified as key barriers to consumption for people living in deprived areas. This relates to both individual income level and the cost of the fish products. Many of the included studies indicated that low income was positively related to a lower amount and frequency of fish consumption (Anderson 2007; Amuzu et al. 2009; Food Foundation 2022; Jones and Chikwama 2021; Kranz et al. 2017; Ruxton 2011). This is supported by evidence that socioeconomic factors and affordability influence fish and seafood consumption overall (Love et al. 2022; DEFRA 2022) including the type of fish item selected. Most of the reviewed studies found that the types of fish eaten in lower income areas were often canned, processed or fried from take‐aways e.g., fish and chips (Fard et al. 2021; Hardcastle and Blake 2015; O'Neill et al. 2004) because they are cheaper than their fresh equivalents (Love et al. 2022). The inability of lower socioeconomic groups to afford to eat a ‘healthy diet’ is well evidenced (Pechey and Monsivais 2016; Scott et al. 2018) with ever increasing reliance on cheap food with lower nutritional value (Food Foundation 2023a) such as processed foods, consumption of which is known to be greater in lower socioeconomic groups (Rauber et al. 2018). This evidence points to healthy eating as a potentially unachievable goal within present social, economic and cultural systems (Carlisle and Hanlon 2003) which likely explains low adherence to ‘healthy eating’ guidance (Scheelbeek et al. 2020), and this is accentuated by the current cost of living crisis (Food Foundation 2023b). Consequently, affordability should be carefully considered alongside other identified barriers to fish intake. There is an urgent need for interventions/campaigns that support communities with advice on buying cheaper fish and how to cook economical fish dishes to suit lower incomes.

#### 5.9.2 Cost

High cost is the most frequently reported barrier for fish intake (Grieger et al. 2012; Govzman et al. 2021). Jones and Chikwama's (2021) study clearly suggested that the ability to buy an expensive protein source such as fish (particularly oily fish), when household costs are rising considerably, is a major population‐level barrier to fish intake. This is supported by the Food Foundation (2023a) who report that the most deprived fifth of adults consume 54% less oily fish than the least deprived fifth. This is not surprising given that retail seafood costs more than other protein foods (Love et al. 2022) although, with the previously reported barriers to fish intake (Ruxton 2011; DEFRA 2022), cost is only one factor. In addition to the urgent need for specific pricing policies, the scoping review findings indicate that to make fresh and oily fish more cost effective for consumers, further emphasis on food systems research, policy and practice is needed. This might take the form of innovative practices, such as promotion of different (lesser known, smaller) fish species which might be more affordable for consumers (Kawarazuka and Béné 2011) and which could enable better alignment and coherence between the blue food system and public health nutrition policies (Koehn et al. 2022).

### 5.10 Strengths and Limitations

This review has strengths relating to the validated framework used to systematically search the databases and clearly map data inclusion, extraction, and collation processes. Furthermore, the inclusion of grey literature is also deemed a strength (Paez 2017), because it permits the published literature to be effectively ‘sense checked’. Our attempt at expert consultations (via email) also supported this, although responses to emails were not forthcoming, and this element could have been strengthened to more robustly meet the sixth component of Arksey and O'Malley's framework (Arksey and O'Malley 2005). Such scoping review expert consultations have been critiqued as not having sufficient standardised guidance on their implementation, so caution is required (Buus et al. 2022). However, having not pursued this element fully, we may have missed other important parts of the grey literature that hold additional data. There are other inherent limitations to this scoping review. This is a complex and nuanced topic; scoping review methodology does not include quality appraisal of studies, which, if carried out, might have permitted some further critique of the topic, particularly in relation to diversity of design aspects, including dietary assessment methods undertaken in the papers (Peters et al. 2021). For example, use of 24 h recall is not sufficiently sensitive to fish intake variations due to prevalence of low intake (Gibson et al. 2017) across SES groups. This method is also subject to under‐reporting (Johansson et al. 2001). Another limitation is that some included papers did not break down their fish intake findings in relation to deprivation specifically, making it difficult to synthesise the data. This required reflexivity (Mak and Thomas 2022) and team discussion to enable transparency and clarity of interpretation and reporting. Similarly, only UK‐based studies were included, although themes may be relevant to other high‐income countries, as some wider literature is discussed. Finally, parameters for the collation and categorisation of studies (Table 1) could perhaps have been better defined.

Despite these limitations, this scoping review provides highly relevant insights to better understand what is known (barriers and drivers) about fish consumption in people living in disadvantaged communities in the UK. This review has already supported benchmarking activities of a nationally important food systems transformation project (FoodSEqual 2021) and hereby provides practical recommendations that can be utilised by researchers, practitioners, and policy makers engaged in blue food system projects, as well as the design of interventions and campaigns to promote fish intake in disadvantaged communities.

## 6 Recommendations for Research, Practice and Policy

- Future research should consider the following:
  ○ Better understanding of the determinants of fish intake across the lifecycle, including younger and older (disadvantaged) communities.
  ○ Focusing on (disadvantaged) minority groups such as males and ethnic groups in relation to their fish intake habits, to improve inclusion and equity.
  ○ Better insights into the lived experience of diverse disadvantaged groups in relation to drivers and barriers to fish intake.
  ○ The importance of the socio‐cultural context of fish‐eating behaviours, particularly in minority groups.
- Future action‐based practice needs to consider the following:
  ○ Facilitate and enable improved knowledge and skills around fish in deprived communities through educational interventions and campaigns designed to upskill people to understand, prepare, and cook fish (and seafood).
  ○ Include advice on affordability and buying cheaper fish species and cooking budget fish dishes.
  ○ Include stakeholders from across the blue food system to engage optimally in education and knowledge exchange on fish.
- Future policies should carefully consider the following:
  ○ The role of wider structural factors in the blue food system (such as the food/shopping environment and supply chain) as drivers and barriers to improved access and intake of fish.
  ○ Context specific pricing policies that should include system stakeholders (e.g., retailers and processors) in their development.

## 7 Conclusion

This scoping review identified several (*n* = 26) published and grey literature studies that inform the evidence base around better understanding the fish intake of people living within disadvantaged communities. Despite some inconsistencies and limitations, findings effectively consolidate the knowledge that people from lower socioeconomic backgrounds are not consuming enough fish (especially oily), compared to government recommendations and that low income and the cost of fish are likely key contributing factors. Similarly, trends are seen that age and sex, as well as (ethnicity and) culture are important determinants of fish‐eating behaviours in lower SES groups. Education level seems to play the most important role, with oily fish consumption increasing by education and occupational levels which impact skills and confidence to prepare and cook it. Thus, there is a need for urgent public health campaigns to improve UK fish intakes. Recommendations are made for research, practice and policy that include (educational) campaigns and strategies to improve skills, knowledge and budgeting, alongside fuller consideration of the wider socio‐cultural and political structural (systems) factors that contribute to low intake of fish in people from disadvantaged communities. A key insight provided by this scoping review is that there is limited evidence enabling understanding of the apparent disparities in determinants of fish intake of people living in disadvantaged communities. There is a particular lack of studies exploring the lived experience of older adults and minority ethnic groups, which reinforces the need for research to be focussed accordingly.

To date, there has been a lack of inclusion of ‘blue foods’ in research debates relating to their system (Tigchelaar et al. 2022) because they are ethically complex food commodities. On the one hand, fish is culturally important and gives us essential nutrients that protect against diseases like cancer and heart disease. In the UK, most people do not eat the recommended amount of fish (two portions a week, one of these portions oily fish) and as this scoping review confirms, socioeconomic status plays an important role. There exists a paradox, however, whereby disadvantaged communities *should* be eating more fish to fulfil nutritional health, yet globally fish stocks are massively depleting. Innovation is required, therefore, to ensure that ‘blue foods’ become a more prominent feature of food system transformation research and action (Nicolini et al. 2024). Our scoping review supports this by making recommendations for research, practice and policy agendas so that better consideration can be given to the importance of socioeconomic status in relation to health inequalities within the (blue) food system.

## Conflicts of Interest

The authors declare no conflicts of interest.

## Data Availability

Data available on request from the corresponding author.

## References

[nbu70030-bib-0001] Åberg, M. A. , N. Åberg , J. Brisman , R. Sundberg , A. Winkvist , and K. Toren . 2009. “Fish Intake of Swedish Male Adolescents Is a Predictor of Cognitive Performance.” Acta Paediatrica 98, no. 3: 555–560. 19006530 10.1111/j.1651-2227.2008.01103.x

[nbu70030-bib-0002] Achterberg, C. , and C. Miller . 2004. “Is One Theory Better Than Another in Nutrition Education? A Viewpoint: More Is Better.” Journal of Nutrition Education and Behavior 36, no. 1: 40. 10.1016/s1499-4046(06)60127-9. 14756981

[nbu70030-bib-0003] Adam, S. J. , L. Goffe , A. J. Adamson , et al. 2015. “Prevalence and Socio‐Demographic Correlates of Cooking Skills in UK Adults: Cross‐Sectional Analysis of Data From the UK National Diet and Nutrition Survey.” International Journal of Behavioral Nutrition and Physical Activity 12, no. 99: 1–13. 10.1186/s12966-015-0261-x. 26242297 PMC4524366

[nbu70030-bib-0004] Adams, J. , F. C. Hillier‐Brown , H. J. Moore , et al. 2016. “Searching and Synthesising ‘Grey Literature’ and ‘Grey Information’ in Public Health: Critical Reflections on Three Case Studies.” Systematic Reviews 5, no. 1: 164. 10.1186/s13643-016-0337-y. 27686611 PMC5041336

[nbu70030-bib-0005] Ahn, J. , M. Kim , C. W. Won , and Y. Park . 2023. “Association Between Fish Intake and Prevalence of Frailty in Community‐Dwelling Older Adults After 4‐Year Follow‐Up: The Korean Frailty and Aging Cohort Study.” Frontiers in Nutrition 10: 1247594. 10.3389/fnut.2023.1247594. 37706211 PMC10497173

[nbu70030-bib-0006] Amuzu, A. , C. Carson , H. C. Watt , D. A. Lawlor , and S. Ebrahim . 2009. “Influence of Area and Individual Lifecourse Deprivation on Health Behaviours: Findings From the British Women's Heart and Health Study.” European Journal of Preventive Cardiology 16, no. 2: 169–173. 10.1097/HJR.0b013e328325d64d. 19242356

[nbu70030-bib-0007] Anderson, A. S. 2007. “Nutrition Interventions in Women in Low‐Income Groups in the UK.” Proceedings of the Nutrition Society 66, no. 1: 25–32. 17343769 10.1017/S0029665107005265

[nbu70030-bib-0008] Antico, A. , M. Tampoia , R. Tozzoli , and N. Bizzaro . 2012. “Can Supplementation With Vitamin D Reduce the Risk or Modify the Course of Autoimmune Diseases? A Systematic Review of the Literature.” Autoimmunity Reviews 12, no. 2: 127–136. 22776787 10.1016/j.autrev.2012.07.007

[nbu70030-bib-0009] Arksey, H. , and L. O'Malley . 2005. “Scoping Studies: Towards a Methodological Framework.” International Journal of Social Research Methodology 8, no. 1: 19–32. 10.1080/1364557032000119616.

[nbu70030-bib-0010] Attree, P. 2005. “Low‐Income Mothers, Nutrition and Health: A Systematic Review of Qualitative Evidence.” Maternal & Child Nutrition 1, no. 4: 227–240. 10.1111/j.1740-8709.2005.00022.x. 16881905 PMC6860959

[nbu70030-bib-0011] Bakre, A. T. , Y. Song , A. Clifford , et al. 2018. “Determinants of Fish Consumption in Older People: A Community‐Based Cohort Study.” Journal of Aging Research and Lifestyle 7: 163–175. 10.14283/jarcp.2018.27.

[nbu70030-bib-0012] Barton, K. L. , W. L. Wrieden , A. Sherriff , J. Armstrong , and A. S. Anderson . 2015. “Trends in Socioeconomic Inequalities in the Scottish Diet: 2001‐2009.” Public Health Nutrition 18, no. 16: 2970–2980. 10.1017/S1368980015000361. 25771827 PMC10271476

[nbu70030-bib-0013] Bates, B. , A. Lennox , and G. Swan . 2010. National Diet and Nutrition Survey: Headline Results From Year 1 of the Rolling Programme (2008/2009). London: Food Standards Agency. https://assets.publishing.service.gov.uk.

[nbu70030-bib-0014] Bennett, G. , L. A. Bardon , and E. R. Gibney . 2022. “A Comparison of Dietary Patterns and Factors Influencing Food Choice Among Ethnic Groups Living in One Locality: A Systematic Review.” Nutrients 14, no. 5: 941. 35267916 10.3390/nu14050941 PMC8912306

[nbu70030-bib-0015] Birch, D. , J. Memery , N. Johns , and M. Musarskaya . 2018. “Stimulating UK Adolescents' Seafood Consumption.” Journal of International Food & Agribusiness Marketing 30, no. 1: 61–69. 10.1080/08974438.2017.1382423.

[nbu70030-bib-0016] Bourlakis, M. , E. Sawyer , and C. Pettinger . 2022. “Mapping Food Supply Chains for UK Disadvantaged Communities: A Focus on Plymouth.” DSI Annual Conference Proceedings: Resiliency and Adaptability for a Better Global Future. https://pearl.plymouth.ac.uk/cgi/viewcontent.cgi?article=1063&context=hp‐research.

[nbu70030-bib-0017] British Nutrition Foundation (BNF) . 2024. Nutrition for Older Adults. https://www.nutrition.org.uk/nutrition‐for/older‐people/.

[nbu70030-bib-0018] Burns, J. L. , A. Bhattacharjee , and G. Darlington . 2024. “The Guelph Family Health Study. Parental Cooking Confidence Is Associated With Children's Intake of Fish and Seafood.” Canadian Journal of Dietetic Practice and Research 85, no. 1: 54–57. 10.3148/cjdpr-2023-012. 37403973

[nbu70030-bib-0019] Buttriss, J. L. 2019. “Making Every Calorie Count.” Nutrition Bulletin 44, no. 2: 174–188. 10.1111/nbu.12384.

[nbu70030-bib-0020] Buus, N. , L. Nygaard , L. L. Berring , et al. 2022. “Arksey and O'malley's Consultation Exercise in Scoping Reviews: A Critical Review.” Journal of Advanced Nursing 78, no. 8: 2304–2312. 10.1111/jan.15265. 35451517 PMC9545832

[nbu70030-bib-0021] Campbell, M. , D. Smith , J. Baird , C. Vogel , and G. Moon . 2020. “A Critical Review of Diet‐Related Surveys in England, 1970‐2018.” Archives of Public Health 78: 66. 10.1186/s13690-020-00447-6. 32699631 PMC7370528

[nbu70030-bib-0022] Carlisle, S. , and P. Hanlon . 2003. “Connecting Food, Well‐Being and Environmental Sustainability: Towards an Integrative Public Health Nutrition.” Critical Public Health 24, no. 4: 405–417. 10.1080/09581596.2013.877580.

[nbu70030-bib-0023] Carlucci, D. , G. Nocella , B. de Devitiis , R. Viscecchia , F. Bimbo , and G. Nardone . 2015. “Consumer Purchasing Behaviour Towards Fish and Seafood Products. Patterns and Insights From a Sample of International Studies.” Appetite 84: 212–227. 25453592 10.1016/j.appet.2014.10.008

[nbu70030-bib-0024] Carstairs, S. A. , L. C. Craig , D. Marais , and K. Kiezebrink . 2017. “Factors Influencing Mothers' Decisions on Whether to Provide Seafood During Early Years' Feeding: A Qualitative Study.” Appetite 108: 277–287. 27737771 10.1016/j.appet.2016.10.010

[nbu70030-bib-0025] Corfe, S. 2018. What Are the Barriers to Eating Healthily in the UK? Social Market Foundation. https://www.smf.co.uk/wp‐content/uploads/2018/10/What‐are‐the‐barriers‐to‐eating‐healthy‐in‐the‐UK.pdf.

[nbu70030-bib-0026] Darling, A. L. , D. J. Blackbourn , K. R. Ahmadi , and S. A. Lanham‐New . 2018. “Vitamin D Supplement Use and Associated Demographic, Dietary and Lifestyle Factors in 8024 South Asians Aged 40‐69 Years: Analysis of the UK Biobank Cohort.” Public Health Nutrition 21, no. 14: 2678–2688. 10.1017/S1368980018001404. 29936916 PMC10260769

[nbu70030-bib-0027] Darmon, N. , and A. Drewnowski . 2008. “Does Social Class Predict Diet Quality?” American Journal of Clinical Nutrition 87, no. 5: 1107–1117. 18469226 10.1093/ajcn/87.5.1107

[nbu70030-bib-0028] Darmon, N. , and A. Drewnowski . 2015. “Contribution of Food Prices and Diet Cost to Socioeconomic Disparities in Diet Quality and Health: A Systematic Review and Analysis.” Nutrition Reviews 73, no. 10: 643–660. 10.1093/nutrit/nuv027. 26307238 PMC4586446

[nbu70030-bib-0029] de Boer, J. , H. Schösler , and H. Aiking . 2020. “Fish as an Alternative Protein–A Consumer‐Oriented Perspective on Its Role in a Transition Towards More Healthy and Sustainable Diets.” Appetite 152: 104721. 32343989 10.1016/j.appet.2020.104721

[nbu70030-bib-0030] DEFRA . 2022. Barriers and Drivers of Seafood Consumption in the UK Evidence Statement.

[nbu70030-bib-0031] Department for Work and Pensions (DWP) . 2024. Review of the UK Material Deprivation Measures. https://www.gov.uk/government/publications/review‐of‐the‐uk‐material‐deprivation‐measures/summary‐review‐of‐the‐uk‐material‐deprivation‐measures.

[nbu70030-bib-0032] Derbyshire, E. 2019. “Oily Fish and Omega‐3s Across the Life Stages: A Focus on Intakes and Future Directions.” Frontiers in Nutrition 6: 165. 10.3389/fnut.2019.00165. 31781570 PMC6861329

[nbu70030-bib-0033] Dorosty, A. , G. Arero , M. Chamar , and S. Tavakoli . 2016. “Prevalence of Sarcopenia and Its Association With Socioeconomic Status Among the Elderly in Tehran.” Ethiopian Journal of Health Sciences 26, no. 4: 389–396. 10.4314/ejhs.v26i4. 27587937 PMC4992779

[nbu70030-bib-0034] Drejerska, N. , and W. Sobczak‐Malitka . 2023. “Nurturing Sustainability and Health: Exploring the Role of Short Supply Chains in the Evolution of Food Systems‐The Case of Poland.” Food 12, no. 22: 4171. 10.3390/foods12224171. PMC10670132 38002228

[nbu70030-bib-0035] Emmett, P. M. , L. R. Jones , and J. Golding . 2015. “Pregnancy Diet and Associated Outcomes in the Avon Longitudinal Study of Parents and Children.” Nutrition Reviews 73, no. 3: 154–174. 10.1093/nutrit/nuv053. 26395341 PMC4586451

[nbu70030-bib-0036] Fard, N. A. , G. de Francisci Morales , Y. Mejova , and R. Schifanella . 2021. “On the Interplay Between Educational Attainment and Nutrition: A Spatially‐Aware Perspective.” EPJ Data Science 10, no. 1: 18. 10.1140/epjds/s13688-021-00273-y.

[nbu70030-bib-0037] Feraco, A. , A. Armani , I. Amoah , et al. 2024. “Assessing Gender Differences in Food Preferences and Physical Activity: A Population‐Based Survey.” Frontiers in Nutrition 11: 1348456. 10.3389/fnut.2024.1348456. 38445208 PMC10912473

[nbu70030-bib-0038] Food Foundation . 2022. The Broken Plate: The State of the Nation's Food System. https://foodfoundation.org.uk/publication/broken‐plate‐2022.

[nbu70030-bib-0039] Food Foundation . 2023a. The Broken Plate: The State of the Nation's Food System. https://foodfoundation.org.uk/publication/broken‐plate‐2023.

[nbu70030-bib-0040] Food Foundation . 2023b. From Purse to Plate: Implications of the Cost‐Of‐Living Crisis on Health. https://foodfoundation.org.uk/sites/default/files/2023‐03/TFF_Cost%20of%20living%20briefing.pdf.

[nbu70030-bib-0041] Food Standards Agency (FSA) . 2022. Eating Well Choosing Better Tracker Survey Wave 8. https://www.food.gov.uk/research/behaviour‐and‐perception/eating‐well‐choosing‐better‐tracker‐survey‐wave‐8‐2022?print=1.

[nbu70030-bib-0042] FoodSEqual . 2021. Food Systems Equality: Co‐Production of Healthy, Sustainable Food Systems for Disadvantaged Communities UKRI Funded Research Programme. https://research.reading.ac.uk/food‐system‐equality/.

[nbu70030-bib-0043] Frąckiewicz, J. , Z. Sawejko , A. Ciecierska , and M. E. Drywień . 2023. “Gender as a Factor Influencing the Frequency of Meat and Fish Consumption in Young Adults.” Roczniki Państwowego Zakładu Higieny 74, no. 4: 373–384. 10.32394/rpzh.2023.0276. 38116797

[nbu70030-bib-0044] Garcia, A. L. , R. Reardon , M. McDonald , and E. J. Vargas‐Garcia . 2016. “Community Interventions to Improve Cooking Skills and Their Effects on Confidence and Eating Behaviour.” Current Nutrition Reports 5, no. 4: 315–322. 10.1007/s13668-016-0185-3. 27882266 PMC5097072

[nbu70030-bib-0045] Gibson, R. S. , U. R. Charrondiere , and W. Bell . 2017. “Measurement Errors in Dietary Assessment Using Self‐Reported 24‐Hour Recalls in Low‐Income Countries and Strategies for Their Prevention.” Advances in Nutrition 8, no. 6: 980–991. 10.3945/an.117.016980. 29141979 PMC5683000

[nbu70030-bib-0046] Godin, K. , J. Stapleton , S. I. Kirkpatrick , R. M. Hanning , and S. T. Leatherdale . 2015. “Applying Systematic Review Search Methods to the Grey Literature: A Case Study Examining Guidelines for School‐Based Breakfast Programs in Canada.” Systematic Reviews 4, no. 1: 138. 10.1186/s13643-015-0125-0. 26494010 PMC4619264

[nbu70030-bib-0047] Gorissen, S. H. M. , and O. C. Witard . 2017. “Characterising the Muscle Anabolic Potential of Dairy, Meat and Plant‐Based Protein Sources in Older Adults.” Proceedings of the Nutrition Society 77, no. 1: 20–31. 10.1017/s002966511700194x. 28847314

[nbu70030-bib-0048] Gov.UK . 2021. Commission on Race and Ethnic Disparities Research Report. https://www.gov.uk/government/publications/the‐report‐of‐the‐commission‐on‐race‐and‐ethnic‐disparities/foreword‐introduction‐and‐full‐recommendations. 10.1136/bmj.n943 33836999

[nbu70030-bib-0050] Govzman, S. , S. Looby , X. Wang , F. Butler , E. R. Gibney , and C. M. Timon . 2021. “A Systematic Review of the Determinants of Seafood Consumption.” British Journal of Nutrition 126, no. 1: 66–80. 10.1017/s0007114520003773. 32967738

[nbu70030-bib-0051] Grieger, J. A. , M. Mille , and L. Cobiac . 2012. “Knowledge and Barriers Relating to Fish Consumption in Older Australians.” Appetite 59, no. 2: 456–463. 10.1016/j.appet.2012.06.009. 22727774

[nbu70030-bib-0052] Haggarty, P. , D. M. Campbell , S. Duthie , et al. 2009. “Diet and Deprivation in Pregnancy.” British Journal of Nutrition 102, no. 10: 1487–1497. 10.1017/s0007114509990444. 19682400

[nbu70030-bib-0053] Hamer, M. , and G. Mishra . 2009. “Dietary Patterns and Cardiovascular Risk Markers in the UK Low Income Diet and Nutrition Survey.” Nutrition, Metabolism, and Cardiovascular Diseases 20, no. 7: 491–497. 10.1016/j.numecd.2009.05.002. 19692214

[nbu70030-bib-0054] Hardcastle, S. J. , and N. Blake . 2015. “Influences Underlying Family Food Choices in Mothers From an Economically Disadvantaged Community.” Eating Behaviors 20: 1–8. 10.1016/j.eatbeh.2015.11.001. 26554510

[nbu70030-bib-0055] Harris, W. 2007. “Omega‐3 Fatty Acids and Cardiovascular Disease: A Case for Omega‐3 Index as a New Risk Factor.” Pharmacological Research 55, no. 3: 217–223. 10.1016/j.phrs.2007.01.013. 17324586 PMC1899522

[nbu70030-bib-0056] Harris, W. S. , N. L. Tintle , F. Imamura , et al. 2021. “Blood n‐3 Fatty Acid Levels and Total and Cause‐Specific Mortality From 17 Prospective Studies.” Nature Communications 12, no. 1: 2329. 10.1038/s41467-021-22370-2 PMC8062567 33888689

[nbu70030-bib-0057] Hergenrader, A. , M. VanOrmer , M. Thompson , et al. 2022. “Assessing the Impact of Socioeconomic Status on Maternal and Cord Serum Omega‐3 Polyunsaturated Fatty Acid Levels.” Current Developments in Nutrition 6: 660. 10.1093/cdn/nzac061.044.

[nbu70030-bib-0144] Heslehurst, N. , E. Cullen , A. C. Flynn , et al. 2023. “Maternal Obesity and Patterns in Postnatal Diet, Physical Activity and Weight Among a Highly Deprived Population in the UK: The GLOWING Pilot Trial.” Nutrients 15, no. 17: 3805. 10.3390/nu15173805. 37686838 PMC10490453

[nbu70030-bib-0058] Holmes, B. A. , and C. L. Roberts . 2011. “Diet Quality and the Influence of Social and Physical Factors on Food Consumption and Nutrient Intake in Materially Deprived Older People.” European Journal of Clinical Nutrition 65, no. 4: 538–545. 10.1038/ejcn.2010.293. 21266981

[nbu70030-bib-0059] Holmes, B. A. , C. L. Roberts , and M. Nelson . 2008. “How Access, Isolation and Other Factors May Influence Food Consumption and Nutrient Intake in Materially Deprived Older Men in the UK.” Nutrition Bulletin 33, no. 3: 212–220. 10.1111/j.1467-3010.2008.00707.x.

[nbu70030-bib-0060] Honkanen, P. , S. O. Olsen , and B. Verplanken . 2005. “Intention to Consume Seafood—The Importance of Habit.” Appetite 45, no. 2: 161–168. 10.1016/j.appet.2005.04.005. 16011859

[nbu70030-bib-0061] Hunt, L. , C. Pettinger , and C. Wagstaff . 2023. “A Critical Exploration of the Diets of UK Disadvantaged Communities to Inform Food Systems Transformation: A Scoping Review of Qualitative Literature Using a Social Practice Theory Lens.” BMC Public Health 23, no. 1: 1970. 10.1186/s12889-023-16804-3. 37821837 PMC10568843

[nbu70030-bib-0062] Huss, L. R. , S. D. McCabe , J. Dobbs‐Oates , et al. 2013. “Development of Child‐Friendly Fish Dishes to Increase Young Children's Acceptance and Consumption of Fish.” Food and Nutrition Sciences 04, no. 10: 78–87. 10.4236/fns.2013.410a012.

[nbu70030-bib-0063] Jamioł‐Milc, D. , J. Biernawska , M. Liput , L. Stachowska , and Z. Domiszewski . 2021. “Seafood Intake as a Method of Non‐Communicable Diseases (NCD) Prevention in Adults.” Nutrients 13, no. 5: 1422. 10.3390/nu13051422. 33922600 PMC8146377

[nbu70030-bib-0064] Joanna Briggs Institute . 2018. The Joanna Briggs Institute Critical Appraisal Tools for Use in JBI Systematic Reviews Checklist for Analytical Clinical Trial Studies. https://jbi.global/critical‐appraisal‐tools.

[nbu70030-bib-0065] Johansson, G. , Å. Wikman , A. Åhrén , G. Hallmans , and I. Johansson . 2001. “Underreporting of Energy Intake in Repeated 24‐Hour Recalls Related to Gender, Age, Weight Status, Day of Interview, Educational Level, Reported Food Intake, Smoking Habits and Area of Living.” Public Health Nutrition 4, no. 4: 919–927. 10.1079/phn2001124. 11527517

[nbu70030-bib-0066] Jones, E. , and C. Chikwama . 2021. “Access to Marine Ecosystems Services: Inequalities in Scotland's Young People.” Ecological Economics 188: 107–139. 10.1016/j.ecolecon.2021.107139.

[nbu70030-bib-0067] Kawarazuka, N. , and C. Béné . 2011. “The Potential Role of Small Fish Species in Improving Micronutrient Deficiencies in Developing Countries: Building Evidence.” Public Health Nutrition 14, no. 11: 1927–1938. 10.1017/S1368980011000814. 21729489

[nbu70030-bib-0068] Khan, S. U. , A. N. Lone , M. S. Khan , et al. 2021. “Effect of Omega‐3 Fatty Acids on Cardiovascular Outcomes: A Systematic Review and Meta‐Analysis.” EClinicalMedicine 38: 100997. 10.1016/j.eclinm.2021.100997. 34505026 PMC8413259

[nbu70030-bib-0070] Koehn, J. Z. , E. H. Allison , K. Villeda , et al. 2022. “Fishing for Health: Do the World's National Policies for Fisheries and Aquaculture Align With Those for Nutrition?” Fish and Fisheries 23, no. 1: 125–142. 10.1111/faf.12603.

[nbu70030-bib-0071] Koehn, J. Z. , E. L. Quinn , J. J. Otten , E. H. Allison , and C. M. Anderson . 2020. “Making Seafood Accessible to Low‐Income and Nutritionally Vulnerable Populations on the U.S. West Coast.” Journal of Agriculture, Food Systems, and Community Development 10, no. 1: 171–189. 10.5304/jafscd.2020.101.027. 33996191 PMC8121265

[nbu70030-bib-0072] Kranz, S. , N. R. Jones , and P. Monsivais . 2017. “Intake Levels of Fish in the UK Paediatric Population.” Nutrients 9, no. 4: 392. 10.3390/nu9040392. 28420147 PMC5409731

[nbu70030-bib-0073] Krauss, R. M. , R. H. Eckel , B. Howard , et al. 2000. “AHA Dietary Guidelines: Revision 2000: A Statement for Healthcare Professionals From the Nutrition Committee of the American Heart Association.” Circulation 102, no. 18: 2284–2299. 10.1161/01.CIR.102.18.2284. 11056107

[nbu70030-bib-0074] Lavelle, F. , L. McGowan , L. Hollywood , et al. 2017. “The Development and Validation of Measures to Assess Cooking Skills and Food Skills.” International Journal of Behavioral Nutrition and Physical Activity 14: 1–13. 10.1186/s12966-017-0575-y. 28865452 PMC5581465

[nbu70030-bib-0075] Lee, H. C. 2022. “Promoting Slow Fish Education in Southern Taiwan Coastal Areas: An Empirical Case Study of Five Elementary Schools.” Marine Policy 138: 104995. 10.1016/j.marpol.2022.104995.

[nbu70030-bib-0076] Lehmann, U. , H. R. Gjessing , F. Hirche , et al. 2015. “Efficacy of Fish Intake on Vitamin D Status: A Meta‐Analysis of Randomized Controlled Trials.” American Journal of Clinical Nutrition 102, no. 4: 837–847. 10.3945/ajcn.114.105395. 26354531

[nbu70030-bib-0077] Lehner, A. , K. Staub , L. Aldakak , et al. 2020. “(2020) Fish Consumption Is Associated With School Performance in Children in a Non‐Linear Way: Results From the German Cohort Study KiGGS.” Evolution, Medicine, and Public Health 1: 2–11. 10.1093/emph/eoz038. PMC6970346 31976073

[nbu70030-bib-0078] Levac, D. , H. Colquhoun , and K. K. O'Brien . 2010. “Scoping Studies: Advancing the Methodology.” Implementation Science 5, no. 1: 1–9. 10.1186/1748-5908-5-69. 20854677 PMC2954944

[nbu70030-bib-0079] Lin, L. Y. , L. Smeeth , S. Langan , and C. Warren‐Gash . 2021. “Distribution of Vitamin D Status in the UK: A Cross‐Sectional Analysis of UK Biobank.” BMJ Open 11, no. 1: e038503. 10.1136/bmjopen-2020-038503. PMC7789460 33408196

[nbu70030-bib-0080] Love, D. C. , A. L. Thorne‐Lyman , Z. Conrad , et al. 2022. “Affordability Influences Nutritional Quality of Seafood Consumption Among Income and Race/Ethnicity Groups in the United States.” American Journal of Clinical Nutrition 116, no. 2: 415–425. 10.1093/ajcn/nqac099. 35691612 PMC9348982

[nbu70030-bib-0081] Macdonald, L. , A. Ellaway , and S. Macintyre . 2009. “The Food Retail Environment and Area Deprivation in Glasgow City, UK.” International Journal of Behavioral Nutrition and Physical Activity 6: 52. 10.1186/1479-5868-6-52. 19660114 PMC2729724

[nbu70030-bib-0082] Maguire, E. R. , and P. Monsivais . 2015. “Socioeconomic Dietary Inequalities in UK Adults: An Updated Picture of Key Food Groups and Nutrients From National Surveillance Data.” British Journal of Nutrition 113, no. 1: 181–189. 10.1017/S0007114514002621. 25399952 PMC4351901

[nbu70030-bib-0083] Mahmudiono, T. , T. S. Nindya , Q. Rachmah , C. Segalita , and L. A. A. Wiradnyani . 2020. “Nutrition Education Intervention Increases Fish Consumption Among School Children in Indonesia: Results From Behavioral Based Randomized Control Trial.” International Journal of Environmental Research and Public Health 17, no. 19: 6970. 10.3390/ijerph17196970. 32977684 PMC7579595

[nbu70030-bib-0084] Mak, S. , and A. Thomas . 2022. “Steps for Conducting a Scoping Review.” Journal of Graduate Medical Education 14, no. 5: 565–567. 10.4300/JGME-D-22-00621.1. 36274762 PMC9580325

[nbu70030-bib-0085] Manietta, C. , M. Rommerskirch‐Manietta , D. Purwins , and M. Roes . 2022. “Consulting Concepts and Structures for People With Dementia in Germany: A Protocol for a ‘Grey‐Shaded’ Scoping Review.” BMJ Open 12, no. 4: e059771. 10.1136/bmjopen-2021-059771. PMC8995961 35396314

[nbu70030-bib-0086] Marinac Pupavac, S. , G. Kenðel Jovanović , Ž. Linšak , M. Glad , L. Traven , and S. Pavičić Žeželj . 2022. “The Influence on Fish and Seafood Consumption, and the Attitudes and Reasons for Its Consumption in the Croatian Population.” Frontiers in Sustainable Food Systems 6: 945186. 10.3389/fsufs.2022.945186.

[nbu70030-bib-0087] Marine Stewardship Council . 2025. What Sustainable Seafood Species Can You Eat in the UK. https://www.msc.org/uk/what‐you‐can‐do/sustainable‐fish‐to‐eat‐in‐the‐uk.

[nbu70030-bib-0088] Marmot, M. 2020. “Health Equity in England: The Review 10 Years on.” BMJ 368. 10.1136/bmj.m693. 32094110

[nbu70030-bib-0089] Martínez‐González, M. A. , A. Gea , and M. Ruiz‐Canela . 2019. “The Mediterranean Diet and Cardiovascular Health: A Critical Review.” Circulation Research 124, no. 5: 779–798. 10.1161/CIRCRESAHA.118.313348. 30817261

[nbu70030-bib-0090] Mesirow, M. S. , C. Cecil , B. Maughan , and E. D. Barker . 2017. “Associations Between Prenatal and Early Childhood Fish and Processed Food Intake, Conduct Problems, and co‐Occurring Difficulties.” Journal of Abnormal Child Psychology 45, no. 5: 1039–1049. 10.1007/s10802-016-0224-y. 27812905 PMC5415431

[nbu70030-bib-0091] Mohan, D. , A. Mente , M. Dehghan , et al. 2021. “Associations of Fish Consumption With Risk of Cardiovascular Disease and Mortality Among Individuals With or Without Vascular Disease From 58 Countries.” JAMA Internal Medicine 181, no. 5: 631–649. 10.1001/jamainternmed.2021.0036. 33683310 PMC7941252

[nbu70030-bib-0092] Monterrosa, E. C. , E. A. Frongillo , A. Drewnowski , S. de Pee , and S. Vandevijvere . 2020. “Sociocultural Influences on Food Choices and Implications for Sustainable Healthy Diets.” Food and Nutrition Bulletin 41, no. 2_suppl: 59S–73S. 10.1177/0379572120975874. 33356592

[nbu70030-bib-0093] Morris, M. C. , D. A. Evans , C. C. Tangney , J. L. Bienias , and R. S. Wilson . 2005. “Fish Consumption and Cognitive Decline With Age in a Large Community Study.” Archives of Neurology 62, no. 12: 1849–1853. 10.1001/archneur.62.12.noc50161. 16216930

[nbu70030-bib-0094] Mozaffarian, D. , R. N. Lemaitre , L. H. Kuller , G. L. Burke , R. P. Tracy , and D. S. Siscovick . 2003. “Cardiac Benefits of Fish Consumption May Depend on the Type of Fish Meal Consumed: The Cardiovascular Health Study.” Circulation 107, no. 10: 1372–1377. 10.1161/01.CIR.0000055315.79177.16. 12642356

[nbu70030-bib-0095] Munn, Z. , D. Pollock , H. Khalil , et al. 2022. “What Are Scoping Reviews? Providing a Formal Definition of Scoping Reviews as a Type of Evidence Synthesis.” JBI Evidence Synthesis 20, no. 4: 950–952. 10.11124/JBIES-21-00483. 35249995

[nbu70030-bib-0096] Neale, E. P. , D. Nolan‐Clark , Y. C. Probst , M. J. Batterham , and L. C. Tapsell . 2012. “Comparing Attitudes to Fish Consumption Between Clinical Trial Participants and Non‐Trial Individuals.” Nutrition & Dietetics 69: 124–129. 10.1111/j.1747-0080.2012.01585.x.

[nbu70030-bib-0097] Nel, L. , P. M. Emmett , J. Golding , and C. M. Taylor . 2025. “Seafood Intake in Children at Age 7 Years and Neurodevelopmental Outcomes in an Observational Cohort Study (ALSPAC).” European Journal of Nutrition 64, no. 3: 1–12. 10.1007/s00394-025-03636-7. PMC11893685 40064696

[nbu70030-bib-0098] Nelson, M. , B. Erens , B. Bates , S. Church , and T. Boshier . 2007. Low‐Income Diet and Nutrition Survey: Volume 2 Food Consumption Nutrient Intake. Foods Standards Agency. https://www.researchgate.net/publication/237525227_Low_income_diet_and_nutrition_survey.

[nbu70030-bib-0099] NHS . 2020. Vitamin D Sources. https://www.nhs.uk/conditions/vitamins‐and‐minerals/vitamin‐d/.

[nbu70030-bib-0100] Nicolini, G. , A. Bladon , J. Clarke , A. Ducros , and A. Guarin . 2024. What About Seafood? The Role of Seafood in UK Food Systems Transformation. IIED and Marine Conservation Society. https://smartthinking.org.uk/report/what‐about‐seafood/.

[nbu70030-bib-0101] Noyeens, A. C. , B. M. van Gelder , H. B. Bueno‐de‐Mesquita , M. P. van Boxtel , and W. M. Verschuren . 2018. “Fish Consumption, Intake of Fats and Cognitive Decline at Middle and Older Age: The Doetinchem Cohort Study.” European Journal of Nutrition 57, no. 4: 1667–1675. 10.1007/s00394-017-1453-8. 28488130

[nbu70030-bib-0102] O'Neill, M. , D. Rebane , and C. Lester . 2004. “Barriers to Healthier Eating in a Disadvantaged Community.” Health Education Journal 63, no. 3: 220–228. 10.1177/001789690406300303.

[nbu70030-bib-0103] Paez, A. 2017. “Grey Literature: An Important Resource in Systematic Reviews.” Journal of Evidence‐Based Medicine 10, no. 3: 233–240. 10.1111/jebm.12265. 28857505

[nbu70030-bib-0104] Parmenter, K. , J. Waller , and J. Wardle . 2000. “Demographic Variation in Nutrition Knowledge in England.” Health Education Research 15, no. 2: 163–174. 10751375 10.1093/her/15.2.163 PMC4344545

[nbu70030-bib-0105] Pechey, R. , and P. Monsivais . 2016. “Socioeconomic Inequalities in the Healthiness of Food Choices: Exploring the Contributions of Food Expenditures.” Preventive Medicine 88: 203–209. 10.1016/j.ypmed.2016.04.012. 27095324 PMC4910945

[nbu70030-bib-0107] Peters, M. D. , C. Marnie , A. C. Tricco , et al. 2021. “Updated Methodological Guidance for the Conduct of Scoping Reviews.” JBI Evidence Synthesis 18, no. 10: 2119–2126. 10.1097/XEB.0000000000000277. 33038124

[nbu70030-bib-0110] Public Health England . 2016. The Eat Well Guide. https://www.gov.uk/government/publications/the‐eatwell‐guide.

[nbu70030-bib-0111] Public Health England . 2020a. The Burden of Disease in England Report. A Report for NHS England. https://assets.publishing.service.gov.uk/media/5e1735f2ed915d3b0b00c7cc/GBD_NHS_England_report.pdf.

[nbu70030-bib-0112] Public Health England . 2020b. NDNS From Years 9–11 Results. https://www.gov.uk/government/statistics/ndns‐results‐from‐years‐9‐to‐11‐2016‐to‐2017‐and‐2018‐to‐2019/ndns‐results‐from‐years‐9‐to‐11‐combined‐statistical‐summary#:~:text=Mean%20consumption%20of%20oily%20fish,less%20than%2020g%20per%20week.

[nbu70030-bib-0113] Rauber, F. , M. L. C. Louzada , E. M. Steele , et al. 2018. “Ultra Processed Food Consumption and Chronic NCD Related Dietary Nutrient Profile in the UK (2008–2014).” Nutrients 10, no. 5: 87. 10.3390/nu10050587. 29747447 PMC5986467

[nbu70030-bib-0114] Rimm, E. B. , L. J. Appel , S. E. Chiuve , et al. 2018. “Seafood Long‐Chain n‐3 Polyunsaturated Fatty Acids and Cardiovascular Disease: A Science Advisory From the American Heart Association.” Circulation 138, no. 1: e35–e47. 10.1161/CIR.0000000000000574. 29773586 PMC6903778

[nbu70030-bib-0115] Romagnolo, D. F. , and O. I. Selmin . 2017. “Mediterranean Diet and Prevention of Chronic Diseases.” Nutrition Today 52, no. 5: 208–222. 10.1097/NT.0000000000000228. 29051674 PMC5625964

[nbu70030-bib-0116] Rondanelli, M. , C. Rigon , S. Perna , et al. 2020. “Novel Insights on Intake of Fish and Prevention of Sarcopenia: All Reasons for an Adequate Consumption.” Nutrients 12, no. 2: 307. 10.3390/nu12020307. 31991560 PMC7071242

[nbu70030-bib-0117] Roos, N. , M. A. Wahab , C. Chamnan , and S. H. Thilsted . 2007. “(2007) the Role of Fish in Food‐Based Strategies to Combat Vitamin A and Mineral Deficiencies in Developing Countries.” Journal of Nutrition 137, no. 4: 1106–1109. 10.1093/jn/137.4.1106. 17374688

[nbu70030-bib-0118] Ruxton, C. H. S. 2011. “The Benefits of Fish Consumption.” Nutrition Bulletin 36, no. 1: 6–19. 10.1111/j.1467-3010.2010.01869.x.

[nbu70030-bib-0119] Scheelbeek, P. , R. Green , K. Papier , et al. 2020. “Health Impacts and Environmental Footprints of Diets That Meet the Eatwell Guide Recommendations: Analyses of Multiple UK Studies.” BMJ Open 10, no. 8: e037554. 10.1136/bmjopen-2020-037554. PMC7451532 32847945

[nbu70030-bib-0120] Scheers, N. , H. Lindqvist , A. M. Langkilde , I. Undeland , and A. S. Sandberg . 2014. “Vitamin B12 as a Potential Compliance Marker for Fish Intake.” European Journal of Nutrition 53, no. 6: 1327–1333. 10.1007/s00394-013-0632-5. 24292746

[nbu70030-bib-0121] Scientific Advisory Committee on Nutrition (SACN) . 2004. Advice on Fish Consumption. UK Government. https://www.gov.uk/government/publications/sacn‐advice‐on‐fish‐consumption.

[nbu70030-bib-0122] Scott, C. , J. Sutherland , and A. Taylor . 2018. Affordability of the UK's Eatwell Guide. Food Foundation. https://foodfoundation.org.uk/publication/affordability‐uks‐eatwell‐guide.

[nbu70030-bib-0123] Soltani, S. , F. Shirani , M. J. Chitsazi , and A. Salehi‐Abargouei . 2016. “The Effect of Dietary Approaches to Stop Hypertension (DASH) Diet on Weight and Body Composition in Adults: A Systematic Review and Meta‐Analysis of Randomized Controlled Clinical Trials.” Obesity Reviews 17, no. 5: 442–454. 10.1111/obr.12391. 26990451

[nbu70030-bib-0124] Sutherland, J. P. , A. Zhou , M. J. Leach , and E. Hyppönen . 2021. “Differences and Determinants of Vitamin D Deficiency Among UK Biobank Participants: A Cross‐Ethnic and Socioeconomic Study.” Clinical Nutrition 40, no. 5: 3436–3447. 10.1016/j.clnu.2020.11.019. 33309415

[nbu70030-bib-0125] Tacon, A. G. , D. Lemos , and M. Metian . 2020. “Fish for Health: Improved Nutritional Quality of Cultured Fish for Human Consumption.” Reviews in Fisheries Science & Aquaculture 28, no. 4: 449–458. 10.1016/j.clnu.2020.11.019.

[nbu70030-bib-0126] Tani, Y. , T. Fujiwara , T. Anzai , and K. Kondo . 2023. “Cooking Skills, Living Alone, and Mortality: JAGES Cohort Study.” International Journal of Behavioral Nutrition and Physical Activity 20: 131. 10.1186/s12966-023-01522-1. 37950296 PMC10636960

[nbu70030-bib-0127] Tigchelaar, M. , J. Leape , F. Micheli , et al. 2022. “The Vital Roles of Blue Foods in the Global Food System.” Global Food Security 33: 100637. 10.1016/j.gfs.2022.100637. 38285816

[nbu70030-bib-0128] Titis, E. , R. Procter , and L. Walasek . 2021. “Assessing Physical Access to Healthy Food Across United Kingdom: A Systematic Review of Measures and Findings.” Obesity Science and Practice 15: 233–246. 10.1002/osp4.563. PMC8976549 35388348

[nbu70030-bib-0129] Tong, T. Y. N. , F. Imamura , P. Monsivais , et al. 2018. “Dietary Cost Associated With Adherence to the Mediterranean Diet, and Its Variation by Socioeconomic Factors in the UK Fenland Study.” British Journal of Nutrition 119, no. 6: 685–694. 10.1017/S0007114517003993. 29553031 PMC5999016

[nbu70030-bib-0130] Tricco, A. C. , E. Lillie , W. Zarin , et al. 2016. “A Scoping Review on the Conduct and Reporting of Scoping Reviews.” BMC Medical Research Methodology 16, no. 1: 1–10. 10.1186/s12874-016-0116-4. 26857112 PMC4746911

[nbu70030-bib-0131] Tsurumaki, N. , S. Zhang , Y. Tomata , et al. 2019. “Fish Consumption and Risk of Incident Dementia in Elderly Japanese: The Ohsaki Cohort 2006 Study.” British Journal of Nutrition 122, no. 10: 1182–1191. 10.1017/S0007114519002265. 31477191

[nbu70030-bib-0132] Umemoto, S. , U. Onaka , R. Kawano , et al. 2022. “Effects of a Japanese Cuisine‐Based Antihypertensive Diet and Fish Oil on Blood Pressure and Its Variability in Participants With Untreated Normal High Blood Pressure or Stage I Hypertension: A Feasibility Randomized Controlled Study.” Journal of Atherosclerosis and Thrombosis 29, no. 2: 152–173. 10.5551/jat.57802. 33298663 PMC8803568

[nbu70030-bib-0133] Utri‐Khodadady, Z. , D. Skolmowska , and D. Głąbska . 2024. “Determinants of Fish Intake and Complying With Fish Consumption Recommendations—A Nationwide Cross‐Sectional Study Among Secondary School Students in Poland.” Nutrients 16: 853. 10.3390/nu16060853. 38542763 PMC10974406

[nbu70030-bib-0134] Verbeke, W. , I. Sioen , Z. Pieniak , J. van Camp , and S. de Henauw . 2005. “Consumer Perception Versus Scientific Evidence About Health Benefits and Safety Risks From Fish Consumption.” Public Health Nutrition 8, no. 4: 422–429. 10.1079/PHN2004697. 15975189

[nbu70030-bib-0135] Wardle, J. , A. M. Haase , A. Steptoe , M. Nillapun , K. Jonwutiwes , and F. Bellisle . 2004. “Gender Differences in Food Choice: The Contribution of Health Beliefs and Dieting.” Annals of Behavioral Medicine 27: 107–116. 10.1207/s15324796abm2702_5. 15053018

[nbu70030-bib-0136] Whybrow, S. , J. L. Hollis , and J. I. Macdiarmid . 2018. “Social Deprivation Is Associated With Poorer Adherence to Healthy Eating Dietary Goals: Analysis of Household Food Purchases.” Journal of Public Health 40, no. 1: e8–e15. 10.1093/pubmed/fdx007. 28158783

[nbu70030-bib-0137] Willett, W. C. , F. Sacks , A. Trichopoulou , et al. 1995. “Mediterranean Diet Pyramid: A Cultural Model for Healthy Eating.” American Journal of Clinical Nutrition 61, no. 6: 1402S–1406S. 10.1093/ajcn/61.6.1402S. 7754995

[nbu70030-bib-0138] Williamson, S. , M. McGregor‐Shenton , B. Brumble , B. Wright , and C. Pettinger . 2017. “Deprivation and Healthy Food Access, Cost and Availability: A Cross‐Sectional Study.” Journal of Human Nutrition and Dietetics 30, no. 6: 791–799. 10.1111/jhn.12489. 28608509

[nbu70030-bib-0139] Worsley, A. 2002. “Nutrition Knowledge and Food Consumption: Can Nutrition Knowledge Change Food Behaviour?” Asia Pacific Journal of Clinical Nutrition 11, no. Suppl 3: S579–S585. 10.1046/j.1440-6047.11.supp3.7.x. 12492651

[nbu70030-bib-0140] Wrieden, W. L. , A. S. Anderson , P. J. Longbottom , K. Valentine , M. Stead , and M. Caraher . 2007. “The Impact of a Community‐Based Food Skills Intervention on Cooking Confidence, Food Preparation Methods and Dietary Choices–an Exploratory Trial.” Public Health Nutrition 10, no. 2: 203–211. 10.1017/S1368980007246658. 17261231

[nbu70030-bib-0141] Wrieden, W. L. , J. Connaghan , C. Morrison , and H. Tunstall‐Pedoe . 2004. “Secular and Socioeconomic Trends in Compliance With Dietary Targets in the North Glasgow MONICA Population Surveys 1986–1995: Did Social Gradients Widen?” Public Health Nutrition 7, no. 7: 835–842. 10.1079/PHN2004636. 15482607

[nbu70030-bib-0142] Yusuf, S. , D. Wood , J. Ralston , and K. S. Reddy . 2015. “The World Heart Federation's Vision for Worldwide Cardiovascular Disease Prevention.” Lancet 386, no. 9991: 399–402. 10.1016/S0140-6736(15)60265-3. 25892680

[nbu70030-bib-0143] Zhu, Y. , J. O. Mierau , I. J. Riphagen , et al. 2023. “Types of Fish Consumption Differ Across Socioeconomic Strata and Impact Differently on Plasma Fish‐Based Omega‐3 Fatty Acids: A Cross‐Sectional Study.” European Journal of Nutrition 63: 435–443. 10.1007/s00394-023-03274-x. 37985508 PMC10899282

